# Glycation‐mediated tissue‐level remodeling of brain meningeal membrane by aging

**DOI:** 10.1111/acel.13805

**Published:** 2023-02-28

**Authors:** Hyo Min Kim, Shinheun Kim, Jueun Sim, Boo Soo Ma, Insung Yong, Youngmin Jo, Taek‐Soo Kim, Jae‐Byum Chang, Sung‐Hye Park, Yong Jeong, Pilnam Kim

**Affiliations:** ^1^ Department of Bio and Brain Engineering Korea Advanced Institute of Science and Technology (KAIST) Daejeon Korea; ^2^ Program of Brain and Cognitive Engineering Korea Advanced Institute of Science and Technology (KAIST) Daejeon Korea; ^3^ Center for Cognition and Sociality Institute for Basic Science (IBS) Daejeon Korea; ^4^ Department of Materials Science and Engineering Korea Advanced Institute of Science and Technology (KAIST) Daejeon Korea; ^5^ Department of Mechanical Engineering Korea Advanced Institute of Science and Technology (KAIST) Daejeon Korea; ^6^ Neuroscience Research Institute, Department of Pathology Seoul National University College of Medicine Seoul Korea; ^7^ KI for Health Science and Technology Korea Advanced Institute of Science and Technology (KAIST) Daejeon Korea; ^8^ Graduate School of Medical Science and Engineering Korea Advanced Institute of Science and Technology (KAIST) Daejeon Korea

**Keywords:** brain meningeal membrane, discoidin domain‐containing receptor 2, extracellular matrix remodeling, glycation, type‐I collagen

## Abstract

Collagen is a prominent target of nonenzymatic glycation, which is a hallmark of aging and causes functional alteration of the matrix. Here, we uncover glycation‐mediated structural and functional changes in the collagen‐enriched meningeal membrane of the human and mouse brain. Using an in vitro culture platform mimicking the meningeal membrane composed of fibrillar collagen, we showed that the accumulation of advanced glycation end products (AGEs) in the collagen membrane is responsible for glycation‐mediated matrix remodeling. These changes influence fibroblast‐matrix interactions, inducing cell‐mediated ECM remodeling. The adherence of meningeal fibroblasts to the glycated collagen membrane was mediated by the discoidin domain‐containing receptor 2 (*DDR2*), whereas integrin‐mediated adhesion was inhibited. A‐kinase anchoring protein 12 (AKAP12)‐positive meningeal fibroblasts in the meningeal membrane of aged mice exhibited substantially increased expression of *DDR2* and depletion of integrin beta‐1 (*ITGB1*). In the glycated collagen membrane, meningeal fibroblasts increased the expression of matrix metalloproteinase 14 (*MMP14*) and less tissue inhibitor of metalloproteinase‐1 (*TIMP1*). In contrast, the cells exhibited decreased expression of type I collagen (*COL1A1*). These results suggest that glycation modification by meningeal fibroblasts is intimately linked to aging‐related structural and functional alterations in the meningeal membrane.

## Graphical abstract

Using the in vitro model of AGE‐modified fibrous collagen membrane, we found the changes in adherence and matrix remodeling function of meningeal fibroblast cells on the glycated membrane. The matrix adhesion of meningeal fibroblasts to the glycated collagen membrane is mediated by the discoidin domain‐containing receptor 2 (DDR2), whereas integrin‐mediated adhesion is inhibited. Also, the glycation‐mediated modification of collagen disrupts the collagen membrane at the brain barrier.

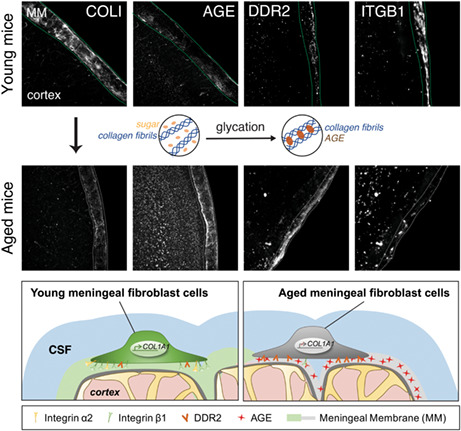

**Abbreviations**
AGE: Advanced Glycation End Product
AKAP12: A‐kinase Anchoring Protein 12
COL1: Type I Collagen
COL4: Type IV Collagen
CSF: Cerebrospinal Fluid
Cx: Cortex
DDR2: Discoidin Domain‐containing Receptor 2
DEG: Differentially Expressed Gene
ECM: Extracellular Matrix
GVC: Glycated vitrified collagen membrane
HMC: Human Meningeal Fibroblast cell
ITGB1: Integrin Beta 1
MM: Meningeal Membrane
MMP14: Metalloproteinase 14
TIMP1: Tissue Inhibitor of Metalloproteinase 1
VC: Vitrified collagen membrane

## 1 INTRODUCTION

Leptomeninges are the inner extracellular matrix (ECM) layers surrounding the brain parenchyma, consisting of arachnoid and pia mater. The leptomeningeal membrane plays role as a protective and supportive barrier for the cerebral cortex against external factors. For example, reaching the cortex of inflammatory cells, bacteria, circulating tumor cells (Weller et al., 2018), and even synthetic macromolecules injected into the cerebrospinal fluid (CSF) (Iliff et al., 2012) are prevented by attaching or filtering the membrane. In this process, the leptomeningeal fibroblasts underlying the pia mater have revealed that they modify the local microenvironment by secreting ECM (Halfter et al., 2002), serving as an efficient physiological barrier against bacterial infection (Wallis & Galyov, 2000; Weller et al., 2018). Although the crucial barrier function of the leptomeninges has been studied extensively, their age‐related changes have attracted little attention.

Aging‐related ECM remodeling is found throughout the body (Horn et al., 2012; Kehlet et al., 2018; Marcos‐Garcés et al., 2014; Schüler et al., 2021), including the brain (McKenna et al., 2021; Reed et al., 2018). Among numerous ECM remodeling mechanisms, glycation that follows the Maillard reaction is one of the best‐known matrix remodeling processes, which leads to the assembly of advanced glycation end products (AGEs) (Goldin et al., 2006). AGEs are formed in a hyperglycemic environment and modify proteins or lipids by a spontaneous nonenzymatic processes. The presence of AGEs has been widely considered as a neurodegenerative process in the brain and a mechanism for aging‐related impairment of cellular function (Kikuchi et al., 2003; Salahuddin et al., 2014). The reduction of CSF turnover rate with aging (Chen et al., 2010) can increase the residence time of AGEs in the CSF and affect AGE‐mediated modification of the meningeal membrane composed of collagen. Although numerous studies have demonstrated the increased concentration of glycated proteins in the aged CSF (Shuvaev et al., 2001), little is known about the effects of AGE formation on the brain meningeal membrane.

AGE‐mediated modification is exceptionally high in long‐lived proteins, such as collagens, that make up 30% of total protein in the body (Ricard‐Blum, 2011). In general, AGEs naturally accumulate along type I collagen (COL1) fibers via crosslinking with lysine‐related residues (Reiser et al., 1992). Although collagen is virtually absent from the brain ECM, the leptomeningeal membrane predominantly consists of fibrillar COL1 (Dorrier et al., 2021), unlike the other basement membranes in the parenchyma, which are composed of nonfibrillar type IV collagen (COL4) (Heck et al., 2007). As the leptomeningeal layer is directly exposed to the CSF, glucose and sugar molecules in the CSF could encourage nonenzymatic modification of the leptomeningeal membrane. In particular, given that glycated collagen accumulates at a rate of 3.7% annually (Corstjens et al., 2008), glycation‐mediated alteration of the leptomeningeal membrane can occur more in the elderly than in younger people. Furthermore, altering the mechanochemical and biochemical characteristics of the membrane by glycation could impair cellular function and gene expression. However, there have been no reports regarding glycation‐related matrix remodeling and its effects on meningeal fibroblasts located along the membrane.

Here, we report glycation of the collagenous matrix of the leptomeningeal membrane in the human and mouse brain. Using the in vitro model of AGE‐modified fibrous collagen membrane, we elucidate the effects of AGE‐modified collagen on meningeal fibroblast–ECM interactions. In particular, we characterize the AGE‐mediated alteration of matrix‐binding receptors and ECM remodeling‐related cellular function.

## 2 RESULTS

### 2.1 AGE‐mediated modification of COL1 in the human leptomeningeal membrane

**FIGURE 1:**
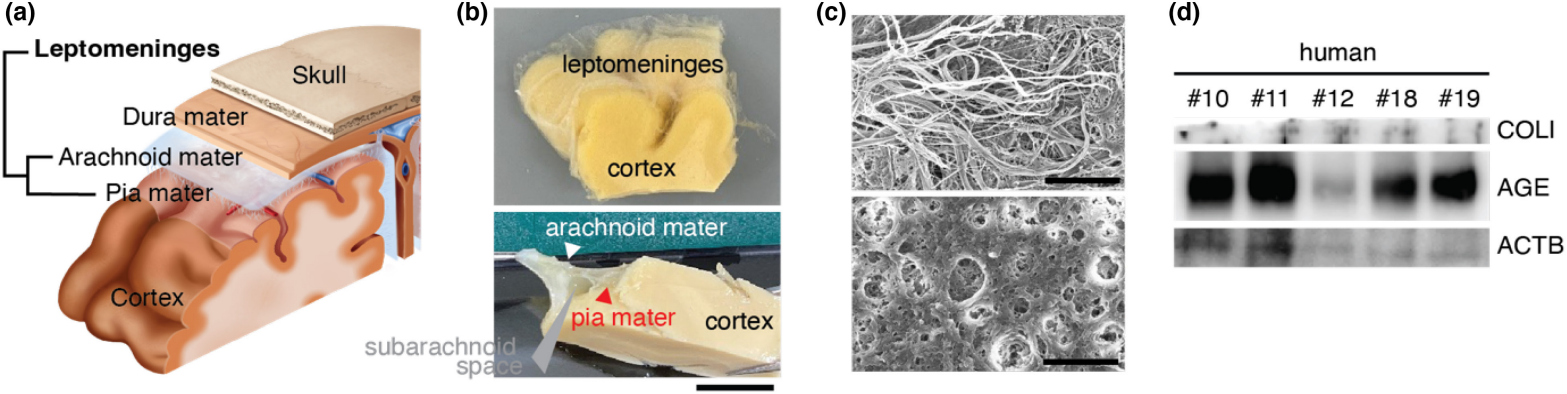
Changes of leptomeninges in the aged human brain. (a) Schematic diagram of leptomeninges in the brain, containing the pia mater and arachnoid mater. (b) The cortex and leptomeninges in the human brain. The thin semitransparent membrane can be distinguished from the cortex (scale bar, 1 cm). (c) SEM image of the fibrillary structure of the leptomeningeal membrane from an 81‐year‐old human brain (upper). Fenestration, which is one of the typical morphological characteristics of the leptomeningeal membrane, is shown in the same brain sample (lower). Scale bar, 10 μm. (d) Western blotting analysis showed a low level of COL1 expression and high level of AGE expression regardless of β‐Actin expression.

Human brain tissues, including the cortex and leptomeninges, were separated from the dura mater and skull (Figure 1a). Semitransparent and meshed structures of leptomeninges are representative of the arachnoid mater. We separated the membrane from the cortex and analyzed the leptomeninges composed of both arachnoid mater and pia mater (Figure 1b). In scanning electron microscopy (SEM) images, the human leptomeningeal membrane showed fibrillary and fenestrated structures, which are characteristic of meninges in the central nervous system (Hutchings & Weller, 1986; Figure 1c). To confirm the existence of glycation in the collagen membrane, we performed Western blotting analysis using the aged human leptomeninges. High AGE levels were detected regardless of age, sex, or cause of death (Figure 1d).

### 2.2 The aged mice leptomeningeal membrane exhibits structural and mechanical alterations

**FIGURE 2:**
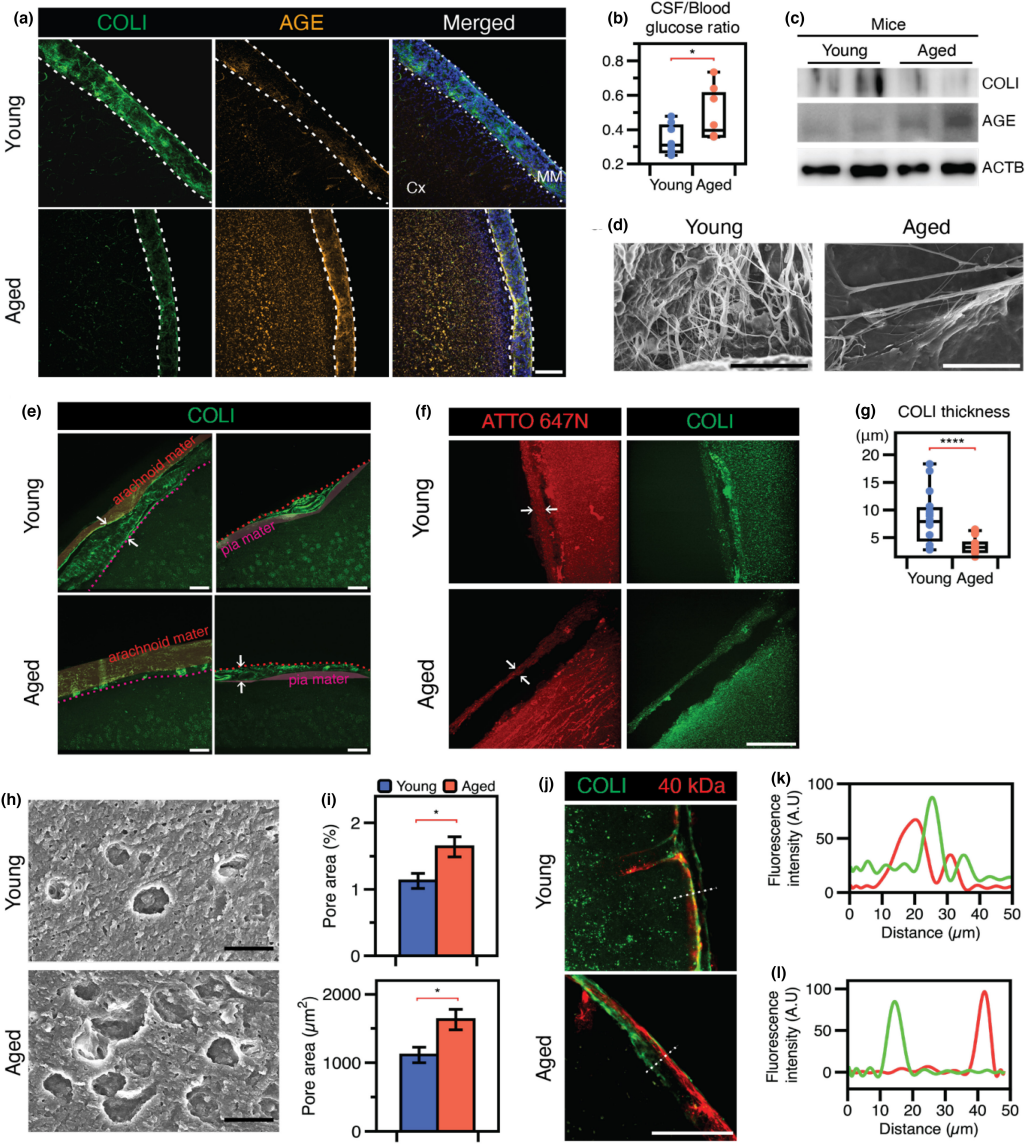
Changes of leptomeningeal membrane in aged mice brain. (a) More COL1 protein with less AGEs in young mice brain than aged mice brain (scale bar, 100 μm). (b) The ratio of glucose levels in CSF and blood is elevated in aged mice. (c) Western blotting analysis confirmed the decrease of COL1 and increase of AGEs. (d) SEM images showing degraded fibrillary structure of leptomeningeal membrane in the aged mice brain (scale bar, 10 μm). (e) Meningeal membranes contain two membranes located both above and below the blood vessels. The meningeal thickness is measured as the width between these two membranes (scale bar, 100 μm). (f) After expansion of brain tissue, another layer of similar thickness is separated from the cortex (scale bar, 100 μm). (g) Meningeal thickness showed reduction in the aged mouse brain. (h) The leptomeningeal membrane of the aged mouse brain has more fenestrated structures than the young mouse brain (scale bar, 10 μm). (i) The size and portion of the fenestrated structures were both increased in the aged leptomeningeal membrane. (j) When 40 kDa dextran molecules were injected into the CSF through the cisterna magna, influx of the tracer through the young leptomeningeal membrane was observed. However, adsorption of the tracer onto the leptomeningeal membrane was observed in the aged mouse brain (scale bar, 100 μm). (k) In the young mouse brain, red fluorescence of 40 kDa dextran molecules was observed on the left of the green fluorescent COL1‐containing membrane. (l) In the aged mouse brain, red fluorescence was seen on the right of the green fluorescent COL1‐containing membrane. Cx, cortex; MM, meningeal membrane. All data are presented as the mean ± SEM. *p < 0.05, **p < 0.01, ***p < 0.001, ****p < 0.0001.

To confirm the AGE‐mediated modification of the leptomeningeal membrane with aging, we compared the meningeal membranes of young mice (younger than 4 months) and aged mice (older than 22 months). Staining for COL1 demonstrated that the pia membrane was present in young and aged mice at the interface. Whereas the membrane in the young mice was continuous and exhibited dense fluorescent signals, that of aged mice showed a discontinuous and loose distribution of collagen. The distribution of AGEs within the membrane was examined using anti‐AGE antibodies that recognized native AGEs. Marked colocalization was observed in both groups of mice, but was significantly enriched in aged mice (Figure 2a), elevating the ratio of glucose level in CSF to blood vessel in the aged mice (Figure 2b).

To further examine AGE‐mediated modification of the collagen membrane, we harvested the leptomeningeal membrane, which is naturally detached from the dura mater membrane (Figures S1), for Western blotting analysis. The expression of COL1 protein is decreased with the elevation of AGEs (Figure 2c). In addition to the changes in the components, we examined structural alteration of the membrane using SEM. The aged membrane shows less fibrillar structure than that of young mice (Figure 2d). Structurally, the leptomeningeal layer in young mice has intact arachnoid mater membrane, consisting of blood vessels between the arachnoid mater and pia mater. In contrast, the leptomeningeal layer of aged mice showed a loose assembly of the covering membrane (Figure 2e). We further performed expansion microscopy to confirm the distinct features of the meningeal membrane. After the expansion process, the layer was separated from the cortex, and the thickness of the layer between young and aged was similar as shown in Figure 2e (Figure 2f). The differences in mechanical properties between the pia membrane and cortex presumably give rise to physical separation under tensorial stress during the expansion process, allowing analysis of the thickness of the leptomeningeal membrane. The membrane was thinner in aged mice than in young mice (3.57 ± 1.51 vs. 8.49 ± 4.56 μm, respectively; Figure 2g). Similar to human brain samples, the meninges of aged mice showed more and larger fenestration structures than those of young mice (Figure 2h,i).

We next investigated whether the highly porous structure of the leptomeningeal membrane affects the barrier function of this layer. We injected 40 kDa dextran conjugated with fluorescent dye as a tracer into the cisterna magna and examined the presence of the dye in the CSF after 1 h. COL1 was used to identify the meningeal layer. The tracer distribution was confirmed by the fluorescent signal from the inside to the outside of the meningeal layer (Figure 2j, white dotted line). In the young mice brain, the tracer was deposited beneath the pial membrane, whereas it was located above the COL1‐expressing membrane in the aged mice (Figure 2k,l). Intracisternal CSF tracer influx through the paravascular space was reduced in aged mice, showing similar to previous observations of reduced peripheral lymphatic transport in aged mice (Kress et al., 2014). The signal intensity at the membrane interface in aged mice was significantly increased compared with young mice for both small (40 kDa) and larger (150 kDa) tracers (Figure S2). Although significant changes have been reported in meningeal lymphatic vessels in both structural and functional impairment associated with aging (Ahn et al., 2019), there was less change in vascular structure between aged and young mice in this study (Figure S3). Therefore, we hypothesized that, with regard to glycation‐mediated changes in the meningeal membrane, the AGE‐mediated modification of the collagen membrane could lead to not only physicochemical changes but also functional impairment related to molecular transport through the membrane.

### 2.3 Fibrillar collagen membrane experiences structural and functional alterations by AGE‐mediated modification

**FIGURE 3:**
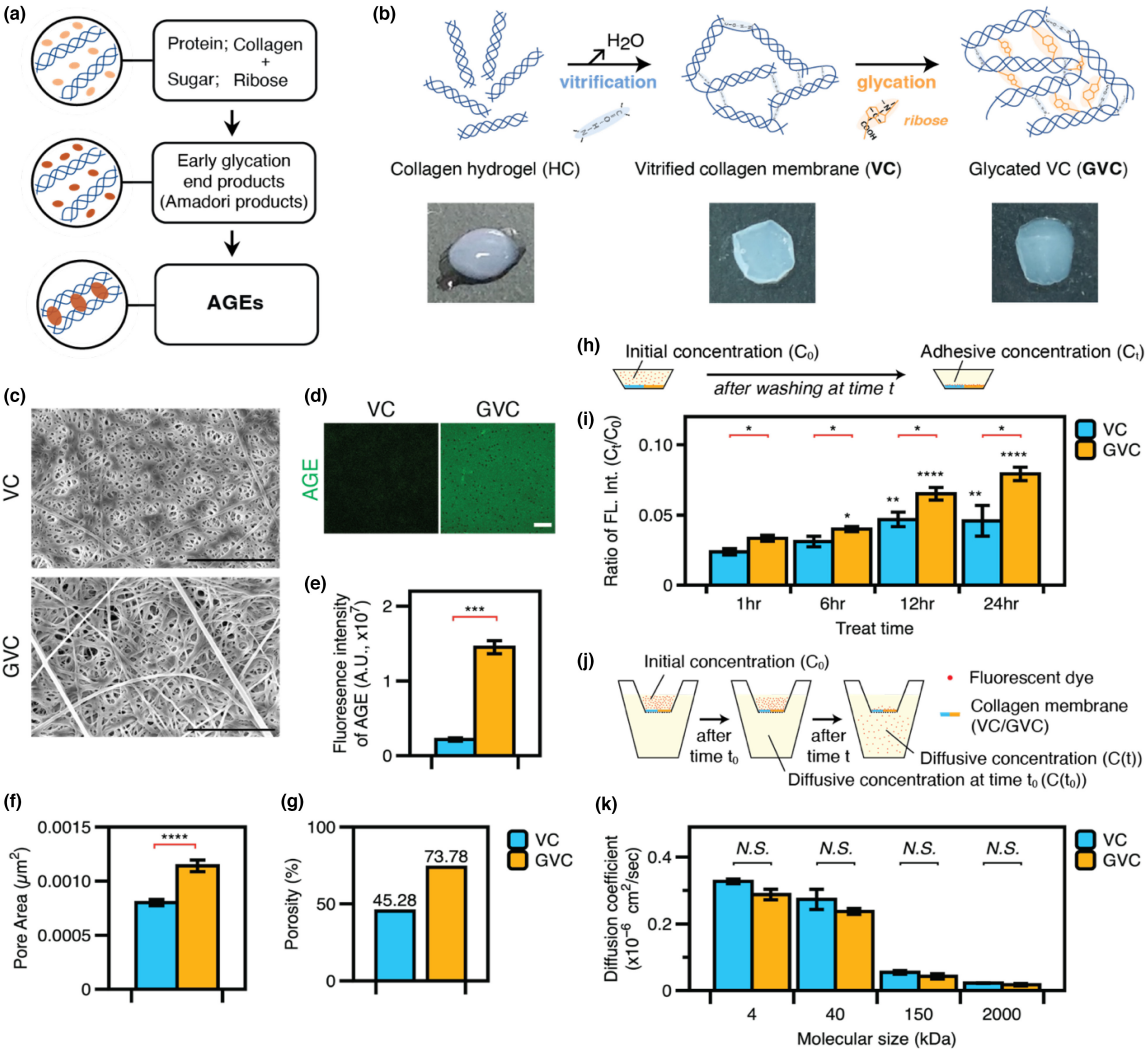
Changes of mechanical function in vitrified collagen membrane by glycation. (a) The glycation process induces AGE accumulation. (b) Schematic diagram showing fabrication of the thin and dense VC and GVC with accumulation of AGEs on its surface. (c) SEM showed that glycation caused collagen fibrils to become porous (scale bar, 10 μm) and fluorescence images showed AGE assembly on the collagen membranes (scale bar, 100 μm). (d, e) Quantitative analysis showed increased pore area which was also validated by porosimetry. (f) Immunofluorescence images of AGE accumulation on the membrane and (g) fluorescence intensity measured by spectrometry were increased by glycation. (h) Experimental scheme for molecular flux through the collagen membrane. (i) Molecular adsorption on the membrane was altered by glycation over time. Asterisks above the bars indicate a significant difference compared with the previous time point on the same membrane. (j) Glycation did not cause molecular diffusion through the membrane. (k) Human meningeal fibroblasts adhered to both collagen membranes and showed meningeal fibroblast cell marker expression (scale bar, 100 μm). *p < 0.05, **p < 0.01, ***p < 0.001, ****p < 0.0001.

To test our hypothesis, we fabricated a fibrillar collagen membrane by vitrification of collagen hydrogel and further induced glycation on the fibrillar collagen membrane (glycated collagen membrane) using D‐ribose (Figure 3a,b). Briefly, a vitrification process was used in which 6 mg/ml of collagen hydrogel became a stiff scaffold after sufficient drying, with a final rehydration process to convert the vitrified matrix to a thickness of 50–100 μm and a transparent membrane with a dense fibrillar structure, resembling the meningeal membrane in vivo (details are presented in the Section 4; Figure 3c).

We first confirmed the AGE‐mediated modification of the collagen membrane based on increased immunofluorescence intensity (Figure 3d) and the autofluorescence signals of AGEs (Hofmann et al., 2013; Figure 3e). The porosity of the collagen membrane was measured by porosimetry. The glycated vitrified collagen membrane (GVC) exhibited increased pore area and porosity compared to the vitrified collagen membrane (VC) (Figure 3f,g). In addition to the structural changes induced by glycation, we examined the changes in physical properties, such as stiffness and adhesiveness, using atomic force microscopy, rheometry, and tensile testing. The glycated collagen membrane showed increased stiffness compared to the non‐glycated membrane in both fibril‐level (Figure S4a–c) and membrane‐level bulk modulus (Figure S4d–h), consistent with research showing that glycation‐mediated collagen crosslinking induces an increase in matrix stiffness (Jang et al., 2022).

Next, to examine the molecular adsorption on the collagen membrane, we treated VC and GVC membranes with fluorescent tracer with a size of 40 kDa (Figure 3h). After washing out the unattached dextran molecules, we measured the fluorescence intensity of the membrane (*C*_t_), and calculated the ratio to the fluorescence intensity of the initial soluble conditions under treatment with dextran molecules (*C*_0_) over time (Figure 3i). The fluorescence intensity ratio in the GVC membrane increased significantly over time compared to the VC membrane, indicating enhanced molecular adsorption at the GVC membrane. In addition, we fabricated VC/GVC membranes on transwell plates with a molecular gradient of fluorescent dye between the insert and the outer well (Figure 3j). We measured fluorescent intensity in the outer well and calculated the diffusion coefficient after 24 h (Figure 3k). Notably, the change in diffusion coefficient through the membrane according to the presence or absence of glycation was not significant but showed molecular size dependency. These results suggest that glycation‐mediated modification enhances molecular adsorption of the collagen membrane, in good agreement with the in vivo observations in the present study.

Taken together, our in vitro model‐based approach provided convincing evidence supporting the intimate relationship between glycation‐mediated AGE accumulation and the structural/functional alterations of the collagen membrane, regardless of cell‐mediated or other aging‐dependent changes in the leptomeningeal membrane.

### 2.4 AGE‐mediated modification of COL1 promotes collagen‐binding receptor tyrosine kinase discoidin domain receptor 2 (DDR2)‐mediated adhesion of meningeal fibroblasts to the ECM but impairs integrin‐mediated adhesion

In parallel, we explored how the aging‐related glycation of the leptomeningeal membrane affects the resident fibroblasts. First, we characterized meningeal fibroblasts to determine the alterations associated with aging using a published single‐cell RNA sequencing (scRNA‐seq) data set (Ximerakis et al., 2019). About 3% of brain cells were *Akap12*‐positive (*Akap12* ^+^) cells and only 7% of these cells expressed *Col1a1* (*Col1a1* ^+^) (Figure S5a). Double positive cells for *Akap12* and *Col1a1* (*Akap12* ^+^/*Col1a1* ^+^) were distinguished from *Col4a1*‐expressing cells (*Akap12* ^+^/*Col4a1* ^+^) (Figure S5b–e). Remarkably, *Col1a1* ^+^ cells were only distributed among the meningeal cells (arachnoid barrier cells, vascular and leptomeningeal cells). In both the young and aged mouse brain, we observed fluorescent signals of AKAP12 and COL1 along the meningeal membrane, with a highly positive correlation between AKAP12 and COL1 (Figure S6a–c). Next, we examined the activation of the receptor for AGEs (RAGE) in cells expressing both AKAP12 and COL1. Immunostaining within leptomeningeal tissues confirmed a typical age‐dependent signal of COL1 but reduced RAGE expression (Figure S7a–c). Consistent with these observations, analysis of the scRNA‐seq data indicated a deficiency of *Ager* gene expression (encoding RAGE) in meningeal fibroblast‐like cells compared to other brain cell clusters (Figure S5f). In general, the effects of RAGE are strongly dependent on cell type and context, even under AGE exposure (Bierhaus & Nawroth, 2009). Therefore, we sought to explore different ligands participating in AGE‐dependent signaling.

**FIGURE 4:**
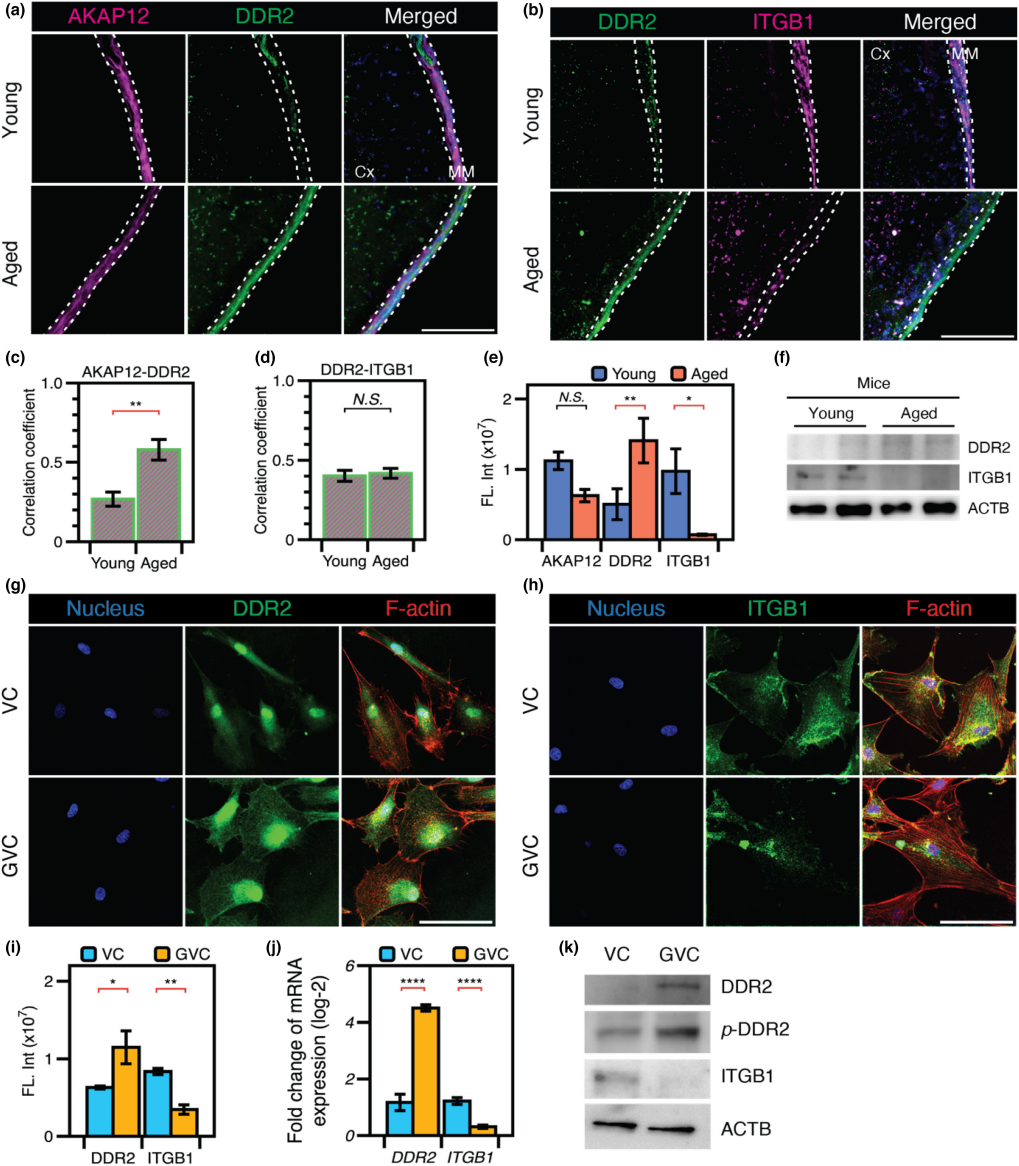
The alternative of ECM‐interactive proteins by aging and glycation. (a) AKAP12‐positive meningeal fibroblasts showed DDR2 expression in the aged mouse brain. (b) ITGB1 depletion was also observed in the aged meningeal membrane. (c) The correlation coefficient between AKAP12 and DDR2 expression was higher in the aged meningeal membrane because the rate of increase in DDR2 expression was much higher than that in AKAP12 expression in the aged mouse brain. (d) In contrast, the correlation coefficient between DDR2 and ITGB1 showed no significant change, suggesting that they do not completely replace each other. (e) While AKAP12 expression was similar (but slightly reduced in the aged mice), the rates of increase in DDR2 and decrease in ITGB1 were much higher in the aged than the young meningeal membrane. (f) These results were confirmed by Western blotting analysis. Increased DDR2 and reduced ITGB1 expression with aging were also seen in HMCs on GVC. (g) As DDR2 is a fibrillar collagen‐binding protein, DDR2 expression was observed in HMCs on both VC and GVC, but DDR2 was expressed over a much broader area on GVC than VC. (h) The depletion of ITGB1 expression was observed on GVC. (i–k) These results were validated by quantitative imaging analysis, PCR, and Western blotting analysis. Cx, cortex; MM, meningeal membrane. Scale bars in all immunofluorescence images, 100 μm. All data are presented as the mean ± SEM. *p < 0.05, **p < 0.01, ***p < 0.001, ****p < 0.0001.

Given that the interactions of the fibrillar collagen matrix with integrins and discoidin domain receptors (DDRs) have been well characterized (Leitinger, 2003), we examined the mRNA expression levels of these genes in meningeal fibroblasts. High levels of Ddr2 expression were seen in aged *Akap12* ^+^ */Col1a1* ^+^ cells (Figure S5g), while aged *Akap12* ^+^ */Col1a1* ^−^ cells showed high levels of Itgb1 expression (Figure S5h). This aging‐dependent expression of adhesion molecules was verified by immunofluorescence staining of DDR2 and ITGB1. In the aged mice, we observed high fluorescence intensity for DDR2 along the AKAP12‐expressing membrane, compared to the low fluorescence intensity of DDR2 in young mice (Figure 4a and Figure S8). Conversely, the aged mice exhibited lower fluorescence intensity of ITGB1 along the DDR2‐expressing meningeal membrane (Figure 4b). The correlation between AKAP12 and DDR2 was significantly increased in aged mice (Figure 4c). In contrast, the correlation between DDR2 and ITGB1 was low in both young and aged mice (Figure 4d). The fluorescence intensities of AKAP12, DDR2, and ITGB1 were quantified in more than four mice per condition (Figure 4e). The fluorescence intensity of AKAP12 showed a slight reduction in aged mice, but the change was not significant. The intensity of labeling for DDR2 was increased, while the intensity of ITGB1 staining was decreased in aged mice. Consistent with the results of immunofluorescence staining, Western blotting analysis showed increased DDR2 and decreased ITGB1 expression in the meningeal membrane of aged mice (Figure 4f).

Next, we examined whether AGE‐mediated modification of collagen matrix remodels the cell‐matrix adhesion in meningeal fibroblasts. We cultured human meningeal fibroblasts (HMCs) on VC versus GVC and observed there is no significant change in morphology and viability (Figure S9). Remarkably, HMCs on GVC showed increased expression of DDR2 in the cytosolic region and reduced expression of ITGB1 compared to cells cultured on VC (Figure 4g,h). The fluorescence intensity was quantified using software FIJI, and the results showed high expression of DDR2 and low expression of ITGB1 in HMCs cultured in the GVC (Figure 4i). Consistent results were obtained by quantitative reverse‐transcription PCR relative to GAPDH (Figure 4j) and by Western blotting analysis (Figure 4k). These observations suggest that AGE‐mediated modification by matrix glycation could promote collagen‐binding receptor tyrosine kinase DDR2‐mediated adhesion of meningeal fibroblasts to the matrix, whereas it could impair integrin‐mediated adhesion.

### 2.5 AGE‐mediated modification of COL1 alters the function of matrix remodeling of meningeal fibroblasts

**FIGURE 5:**
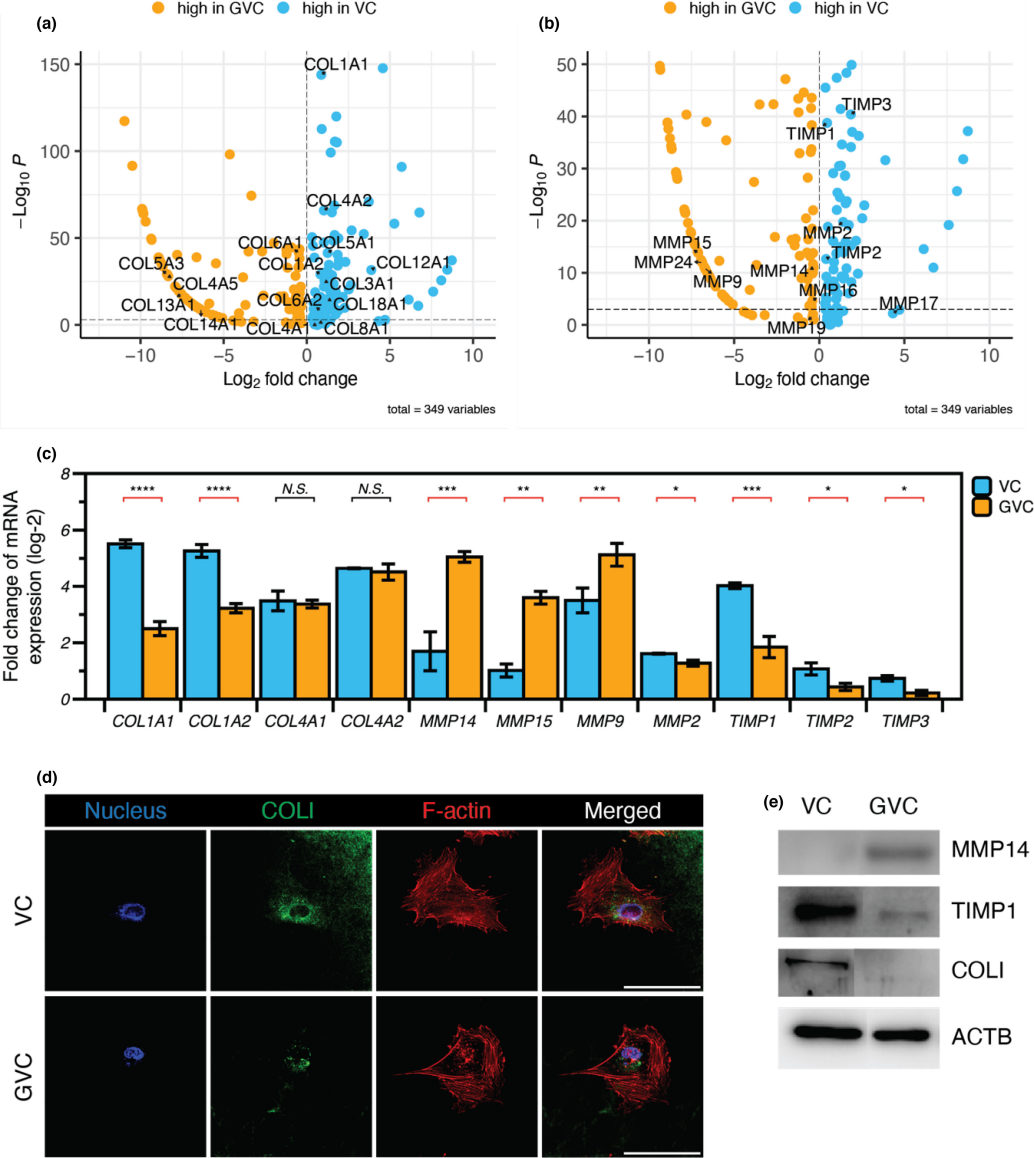
Changes in collagen synthesis and degradation by glycation. (a) In bulk RNA sequencing, we drew volcano plots with ECM‐related genes that showed changes in HMCs in comparison between VC (blue dots) and GVC (orange dots). *COL1A1* expression converted to high significance (*p*‐value) with low fold change between VC and GVC. (b) Several MMPs were highly expressed in HMCs on GVC and TIMP was highly expressed in VC. (c) COL1, including COL1A1 and COL1A2, was reduced on GVC, while COL4, including COL4A1 and COL4A2, was not. This was because of elevated expression of membrane‐bound MMPs (MMP14 and MMP15) and MMP9. In addition, TIMP expression was reduced on GVC, accelerating matrix degradation. (d) COL1 expression in the HMCs was detected by immunofluorescence analysis (scale bar, 100 μm). (e) Western blotting analysis demonstrated increased MMP14 expression and reduced TIMP1 expression following depletion of COL1A1. All data are presented as the mean ± SEM. *p < 0.05, **p < 0.01, ***p < 0.001, ****p < 0.0001.

We performed bulk transcriptomic analysis to examine how glycation‐modified collagen further impacts the function of meningeal fibroblasts. We preprocessed raw data to align bulk RNA‐seq using STAR (detailed in Section 4) and identified differentially expressed genes (DEGs), especially ECM‐related gene sets (Table S5). Among collagen‐related gene sets, COL1A1 showed a significant change with a 1.5‐fold increase in the VC (Figure 5a). The expression of tissue inhibitor of metalloproteinases (TIMP) was markedly upregulated in VC (Figure 5b). Among the matrix metalloproteinases (MMPs), *MMP14* and *MMP15*, which are membrane‐bound types, were significantly increased in GVC. These results were further validated by quantitative PCR (Figure 5c). The observations suggest that collagen degradation in the glycated matrix could be related to the MMP14 activation and reduction of TIMP1 expression. We also observed the decrease in COL1 synthesis on GVC by immunofluorescence analysis and the reduction of *COL1A1* and *COL1A2* gene expression (Figure 5d,e). Taken together, our observations imply that matrix glycation leads to MMP14‐mediated collagen degradation and reduction of TIMP expression, as well as the reduction of collagen synthesis. These results suggest that matrix glycation could cause aging‐related collagen depletion on the brain leptomeningeal membrane, following alteration of collagen remodeling function in meningeal fibroblasts.

## 3 DISCUSSION

**FIGURE 6:**
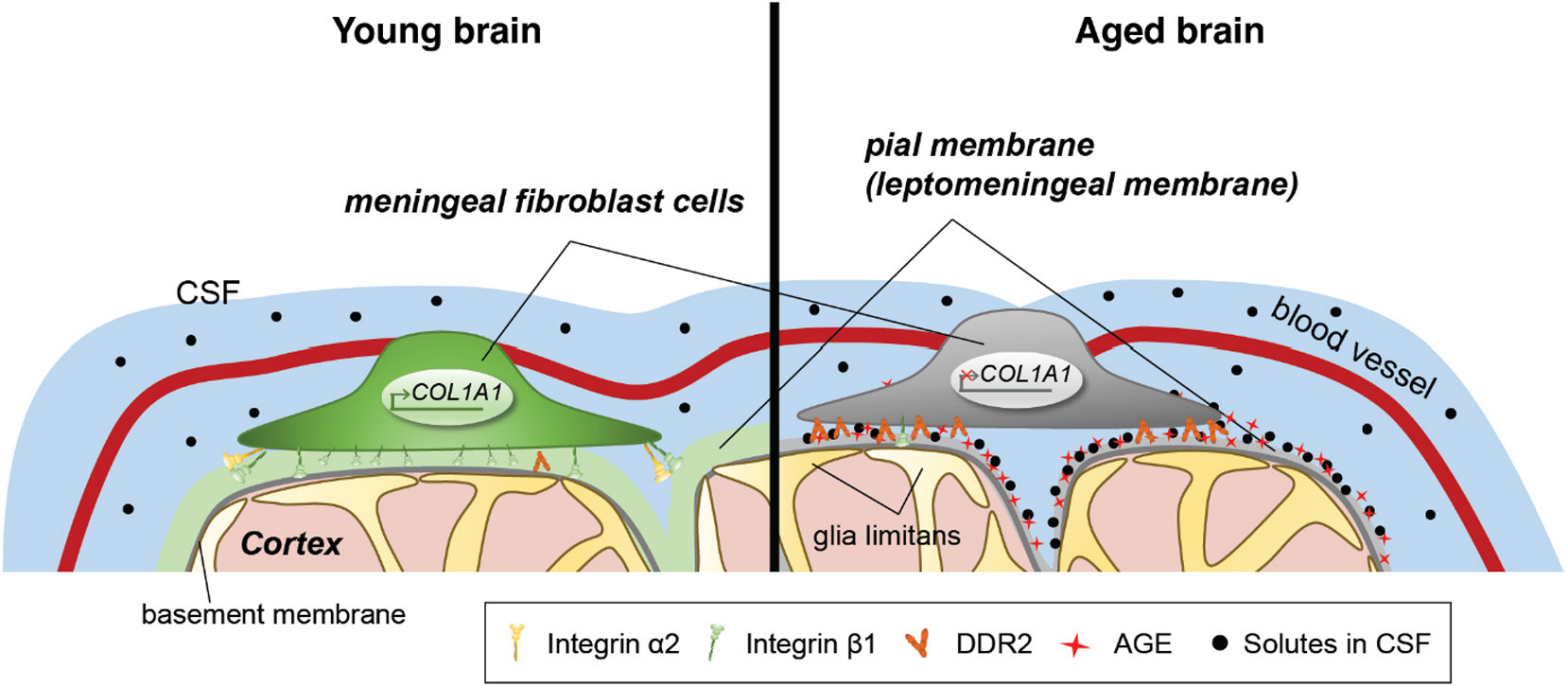
Schematic diagram of proposed mechanism showing aging‐related ECM remodeling through meningeal fibroblasts on the brain leptomeninges. Meningeal fibroblasts in the young brain showed dynamic COL1A1 synthetic and COL1‐interactive function on the collagen membrane. They showed ITGB1‐mediated adhesion on the COL1‐composed leptomeningeal membrane and induction of COL1A1 synthesis for maintaining the collagen membrane. With aging, meningeal fibroblasts showed depletion of COL1A1 synthetic function and altered cell–matrix interaction.

Our results demonstrated the matrix remodeling on the leptomeningeal membrane by an aging‐related process (Figure 6). The aged leptomeninges exhibited matrix glycation that induce alteration of cellular function and adhesion of meningeal fibroblasts. Although hallmarks of neurodegenerative and inflammatory environments, there have been no systematic studies of functional variation associated with these changes (Brown et al., 2018; MacLullich et al., 2004; Shen et al., 2021; Voevodskaya et al., 2014; Wuerfel et al., 2008). Combining the public scRNA‐seq data and the results of our in vitro and in vivo experiments, we confirmed that matrix glycation as an aging‐related process induces alteration of cellular function and adhesion of meningeal fibroblasts.

Despite the importance of the meninges is emerged as a brain barrier system, little is known about the changes in matrix associated with normal aging in terms of physiological and histological modifications. Our study demonstrated that the loss of COL1 by aging is associated with MMP14‐mediated matrix degradation. In addition, the reduction of collagen synthesis is accompanied by altered cell‐matrix interaction of meningeal fibroblasts in the glycation‐modified membrane. Furthermore, we found that the adhesion of meningeal fibroblasts to the glycation‐modified matrix is mediated through DDR2 rather than the integrin receptor. Among the tyrosine kinase receptors *Ddr1* and *Ddr2*, only *Ddr2* showed elevated gene expression in the meningeal fibroblasts of the aged mice brain (Figure S5g). This increase in *Ddr2* expression is consistent with the observation in the meningeal fibroblasts of the aged mice brain (Figure 4a and Figure S8).

Our results also confirmed a major interaction of fibroblasts and meningeal matrix by DDR2 activation as a multiligand receptor response to the glycated environment instead of RAGE expression. In fact, the expression of RAGE is elevated in inflammatory and neurodegenerative diseases (Origlia et al., 2009; Palanissami & Paul, 2018); therefore, RAGE has been a therapeutic target in neurodegenerative diseases (Bongarzone et al., 2017). Despite the fact that RAGE has been studied in the human brain for about 20 years (Li et al., 1998) and there is evidence that AGEs disrupt brain homeostasis (Rom et al., 2020), little is known about the effects of AGEs on the brain meningeal membrane. Based on our observations and public scRNA‐seq data, we uncovered glycation‐mediated activation of DDR2 in meningeal fibroblasts, suggesting that DDR2‐related cellular processes could be associated with aging‐related alteration of the leptomeningeal membrane. DDR2 activation in meningeal fibroblasts may not only represent the remodeling of collagen‐binding motifs but also lead to the alteration of intracellular signaling pathways (Cole et al., 2018; Lemmon & Schlessinger, 2010). Given that cardiac and dermal fibroblasts have been reported increased integrin‐mediated matrix interaction by glycation (Chen et al., 2022; Talior‐Volodarsky et al., 2015), they have RAGE‐dependent responses to the glycated environment (Varani et al., 2006; Zhao et al., 2014). However, adventitial fibroblasts around the vascular structures contribute to ECM production in a RAGE‐independent manner (Kennon & Stewart, 2021). As meningeal fibroblasts showed no RAGE expression even on the glycated collagen membrane and in the aged mice brain, they could occur RAGE‐independent adaptation to the glycated environment, accoupling impairment of integrin‐mediated binding.

Lastly, we found the reduction of collagen levels in the brain meningeal membrane and suggest the alteration in matrix remodeling function of meningeal fibroblasts, classifying as both COL1‐ and AKAP12‐expressing cells. As resident fibroblasts in the meningeal membrane play roles in response to vascular injury (Kuwabara & Tallquist, 2017) and in the glymphatic system (Dorrier et al., 2022), it is important to understand the cellular change in the aging or degenerative process. There are still limitations of understanding the relationship of DDR2 and COL1 and its association with a neurological disease by meningeal fibroblast‐mediated matrix remodeling via glycation. Further studies are required to clarify the aging‐related matrix remodeling of the brain meningeal membrane due to glycation‐adapted fibroblasts. This may provide clues that understanding of aging represents a new target for antiaging research or in the treatment of neurodegenerative diseases.

## 4 EXPERIMENTAL SECTION/METHODS

### 4.1 Preparation of human brain

Post‐mortem human brain samples were provided by Dr. Sung‐Hye Park of Seoul National University Hospital Brain Bank (SNUHBB, Seoul, Korea) containing the area within the meningeal structure. Fresh frozen post‐mortem human brain samples were used for IHC images by sectioning of thickness 10 μm. Frozen human brain tissue was embedded 30% sucrose/OCT (1:1 w/w) solution for 4 h at room temperature (RT) and replaced into OCT solution, freezing in isopentane at liquid nitrogen. The OCT‐embedded tissue samples were stored in deep freezer until sectioning, and formalin‐fixed human brain samples were used for morphology analysis, storing in the 30% sucrose solution until experiments. Formalin‐fixed human brain samples with variety of age, sex, and cause of death were kindly provided by Dr. Im‐Joo Rhyu of College of Medicine in Korea University (Seoul, Korea), and patient information is provided in Table S1. The experiment is exempt from deliberation by the Institutional Review Board.

### 4.2 Animals and surgery

C57BL/6NHsd mice were purchased from Koatech. Both male and female mice at 2–4 months were used as young, and at 20–24 months were used as aged mice. Each experiment was performed with at least four mice, and sex balance was maintained between young and aged conditions. All mice were housed under a 12 h light/dark cycle, and food and water were freely accessible. All procedures were approved by the KAIST Institutional Animal Care and Use Committee (KAIST‐IACUC). Animal care and handling were performed in accordance with their guidelines. In all experiments, animals were anesthetized by intraperitoneally injection with a combination of ketamine (100 mg/kg) and xylazine (20 mg/kg). To expose the cisterna magna, position the head and the neck at a 120° angle with the head fixed to the stereotaxic frame (Model 940, KOPF). After the neck skin incision, the central space between the muscles of both necks through forceps to expose the cisterna magna.

### 4.3 Intracisternal chemical/drug injection and CSF collection

Fluorescent dextrans were purchased from Sigma‐Aldrich (FD4; *4kDa‐FITC*, FD40; *40kDa‐FITC*, 46946; *150kDa‐FITC*, FD2000; *2000kDa‐FITC*, T1287; *150kDa‐TRITC*) Invitrogen (D1842; *40kDa‐Rho*), and TdB Labs (ADR4; *4kDa‐Antonia Red*) to avoid charge effect. The tracers were diluted in ACSF solution (3525, R&D Systems) in 1 mg/ml concentration and 31‐gauge needle was connected with a syringe (84854, Hamilton) using PE 10 tube (#BB31695‐PE/1, Scientific Commodities Inc.) and delivered to the cisterna magna. A total 5 μl was delivered at a rate of 1 μl/min for 5 min by an automatic syringe pump (KD Scientific). Intracisternal delivery and surgery procedure followed Nedergaard's protocol (Xavier et al., 2018). In this paper, Actinomycin D (A9415, Sigma‐Aldrich) was used as in vivo tyrosine kinase inhibitor with concentration 0.25 mg/kg were dissolved in DMSO. Actinomycin D was intraperitoneally injected for 5 days, and DMSO was used as a vehicle control. Five days after injection, tracers were injected into the cisterna magna on Day 7 to confirm the change in CSF influx. To measure the glucose level in the CSF, CSF was collected after puncturing the cisterna magna with a customized glass capillary. CSF was collected as a clear liquid, and the glucose level was measured by a blood glucose meter (AGM 513 S, DW Medipharm). Glass capillaries (1B120‐3, World Precision Instruments) were customized to have a tip diameter 50 μm using micropipette puller (P‐1000, Sutter Instrument).

### 4.4 Cell culture

Human meningeal fibroblast cells (HMCs) isolated from human leptomeninges were purchased from ScienCell Research Laboratories (#1400). Specialty Medium (#1401, ScienCell) was used for culture medium for cells, adding the 2% of fetal bovine serum (FBS), 1% of antibiotic solution (P/S), and 1× meningeal cell growth supplement (MCGS). Cells were cultured in PLL (2 μg/cm^2^) and type‐I collagen (0.1 mg/ml)‐coated T75 flask incubating with 5% CO_2_/95% air. 0.025% Trypsin/EDTA solution was used to detach the cells for experiments and seeding density was 5000 cells/cm^2^, subculturing when the culture reaches 90%–95% confluency. Culturing cells were followed by instructions from ScienCell until passage number 7. To adapt cells with type‐I collagen, T75 flasks were coated with 2 μg/cm^2^ of poly‐L‐lysine solution adding 0.1 mg/ml type‐I collagen diluted in 0.1% acetic acid solution. Cells were seeded at a density of 5000 cells/cm^2^ and cultured for 3 days for all in vitro experiments.

### 4.5 Preparation of vitrified collagen membrane

High‐concentrated type‐I collagen from rat tail was purchased from Corning (354249). 6 mg/ml collagen solution was placed on the cover glasses (40 μl for 96 well plate, 120 μl for 15 pi cover glass, 300 μl for 25 pi cover glass) processed with protein‐binding coating method. After incubating at 37°C at least for 1 h, they were dehydrated in controlled chamber (temperature 20–25°C, humidity 50%–60%) for 3 days and remove extra salts in the collagen membrane by washing by D.W. and PBS, respectively. In addition then, they were dehydrated again in controlled chamber for 1 day more. Glycation was processed by 0.25 M ribose solution in 37°C incubator for 1 week. The collagen membranes were incubated in DPBS until using in experiments. 96‐well plates were used for viability test, drug screening test, and matrix remodeling test; 15 pi cover glasses were used for IF and qPCR experiments; and 25 pi cover glasses were used for Western blot. For binding vitrified collagen membrane on the culture plates or glasses, the surface was first activated with 1 N NaOH for over 4 h at 60°C. Then, they were treated with 1% polyethyleneimine (PEI) solution and 0.1% glutaraldehyde (GA) solution for 1 h each at RT. They were washed with D.W. for at least 2 times between all steps and washed with DPBS just before placing the collagen solution on the plates. To fabricate fluorescent dye‐conjugated collagen vitrigel membrane, we added 5 mg/ml of COL‐F collagen‐binding reagent (ICT‐6346, ImmunoChemistry Technologies) to fabrication step of collagen hydrogel.

### 4.6 Characterization of vitrified collagen membrane

To measure tensile modulus of vitrified collagen membrane, the pseudo free‐standing tensile testing system (Kim et al., 2013) in laboratory of prof. Taek‐Soo Kim was used. PDMS‐attached grips were coated with PEI‐GA to grasp the ultra‐thin membrane and give the frictional drag force on the floating state. The shear modulus was measured by rheometer (MCR92, Anton Paar) under the angular frequency variation from 100 rad/s to 1 rad/s and shear modulus was calculated as $\sqrt{{\text{Storage Modulus}}^{2}+{\text{Loss Modulus}}^{2}}$. Atomic force microscopy (AFM) was performed by Scanning Probe Microscope (XE‐100, Park Systems) in KAIST NanoFab and Field Emission Scanning Electron Microscope (Magellan400, FEI Company) in KAIST Analysis center for Research Advancement (KARA). Sample preparation for SEM image was performed by gradual dehydration using ethyl alcohol from 50%, 70%, 90%, and 100% concentration for 10 min per wash and then placed on the SEM grid directly with carbon tape. Then, samples were sputter coated with Pt with 5–10 nm thickness and observed under 5–10 kV beam voltage. Pore size and istribution of membrane were measured by porosimeter (AutoPore IV 9500, Micromeritics Instrument Corporation) in Hanbat University (RIC Center, Daejeon, Korea) with evacuation pressure 50 μmHg for 5 min and mercury filling pressure 0.52 psia for equilibration time 10 s.

### 4.7 Macromolecular diffusion and adhesion assay

To assay the molecular characterization of membrane, vitrified collagen membrane was fabricated on the transwell membrane inserts (353097, Corning). The apical sides of chambers were filled with DPBS containing various size of fluorescent dextran. The molecular adsorption on the membrane was measured in fluorescence intensity by spectrometer and calculated the ratio of adhesive molecular concentration to the initial molecular concentration: $\frac{C_{t}}{C_{0}}$, where $C_{t}$ is a measured optical density of adhesive fluorescent molecules on the membrane after washing out while adhesion time *t* has given. $C_{0}$ is a measured optical density of initially treated fluorescent molecules on the membrane. We detected fluorescence intensity taken after 1, 6, 12, and 24 h.

Then, absolute permeability *P* [cm/s] was calculated by the following equation: $P=\frac{\left[C\left(t\right)-C\left(t_{0}\right)\right]∙V}{A∙t∙C_{0}}$. *C*(*t*) is a concentration (μg/ml) of fluorescent dextran in the samples from the lower compartment throughout the transwell membrane (A, 0.3 cm^2^) after time t (24‐h in here) with volume *V*, *C* $\left(t_{0}\right)$ is the concentration (μg/ml) of dextran after time 0 h (with washing out right after molecular treatment) and *C*_0_ is the initial concentration (μg/ml) of the dextran (without washing out) on the donor side. The amount of fluorescence for dextran was all measured at specific excitation and emission wavelengths of fluorescent dyes.

### 4.8 Characterization of cell behavior

Cell proliferation assay was utilized with wst‐8 cell viability assay kit (Quanti‐Max, Biomax). The method of assay was water‐soluble tetrazolium salt method to evaluate the biocompatibility of vitrified collagen membrane and glycation effect. The Quanti‐Max solution was added to cell media with 1:10 diluted concentration. Cells on the collagen membrane were incubated with wst‐8 assay solution for 2–3 h. After formation of formazan crystals because of mitochondrial dehydrogenases of cells, the color of cell medium was measured by absorbance using a microplate reader at 450 nm wavelength. The rates of proliferation were measured using the value of optical density (O.D.) and compared to non‐reacted wst‐8 assay solution which was added Quanti‐Max solution into cell media without cells. The value of O.D. was relatively calculated as a normalized fold‐change along the time‐variant. Drug screening tests were performed 1 day after cell seeded. Drugs were treated for 2 days, and the lists were actinomycin D (10 μM, A9415, Sigma‐Aldrich), dasatinib (1 nM, S1021, Selleckchem), and FPS‐ZM1 (250 nM, ab235552, Abcam). The usage was followed from each protocol the company provided. Matrix remodeling test was performed using recombinant MMP proteins which are MMP1 (10532‐H08H, Sino Biologicals), MMP9 (10327‐H08H, Sino Biologicals), and MMP14 (918‐MP‐010, R&D Systems). The concentration of MMP proteins were all used as 0.1 ng/μl. These proteins were treated on the membrane for 3 days just after attached on the membrane when cells were seeded on.

### 4.9 Immunofluorescence imaging

After all experiments, cardiac perfusion was performed with PBS followed by 4% PFA/PBS solution (Au‐Gage et al., 2012). Perfused brains were fixed at 4% PFA for overnight and were stored in 20% sucrose solution during overnight. Then, mice brains were cut into 100 μm thickness of coronal sections using a vibratome (VT1200, Leica Biosystems). For in vitro experiments, HMCs were fixed by 4% PFA for 30 min at RT and washed three times with 1× PBS. The fixed brain slices and cells were permeabilized with a blocking solution containing 5% normal goat serum (NGS) (31872, Thermo Fisher Sceintific) in 1% Triton X‐100/1X PBS (1% PBST) solution for over 1 h at RT. Samples were incubated overnight at 4°C with primary antibody diluted in the blocking solution. Then, samples were washed with PBS three times for 5 min each and incubated for 8–12 h at 4°C with secondary antibody also diluted in the blocking solution. After washing with PBS 3 times for 5 min each, samples were mounted with mounting medium containing DAPI (H‐1200, Vectashield). Antibodies used in this research were listed in Table S2. The images were acquired with confocal microscopes (Plan‐Apochromat 20X/0.8M27, LSM‐880, Carl Zeiss; Plan‐Apochromat 20X/0.75 NA, 40X/0.95 NA, Nikon A1, Nikon). Images were taken with Z stacks in 1–2 μm steps (recommendation by pinhole size) and then merged into a single image by the maximum projection. Brain images to examine CSF influx were acquired through a whole slide imaging system (X10 lens, Axio Scan Z1, Zeiss).

### 4.10 Expansion microscopy imaging

Mice brain slices were prepared for expansion microscopy imaging after antibody incubation as stated previously. The brain slices were washed three times with 0.1% PBST for 30 min each time. Then, the slices were incubated for 6 h with acryloyl‐X at a concentration of 0.1 mg/ml diluted in 1X PBS and then washed three times with 1× PBS. For gelation, the slices were first incubated with a monomer solution (7.5% sodium acrylate, 2.5% acrylamide, 0.15% N,N′‐methylenebis(acrylamide), 2 M NaCl in 1× PBS) twice at 4°C for 30 min each time, and incubated with a gelation solution (8.625% sodium acrylate, 2.5% acrylamide, 0.15% N,N′‐methylenebis(acrylamide), 0.2% ammonium persulfate, 0.2% tetramethylethylenediamine, 0.01% 4‐hydroxy‐2,2,6,6‐tetramethylpiperidin‐1‐oxyl, 2 M NaCl in 1× PBS) at 4°C for additional 30 min. After incubation, inter‐digestion staining of fluorophore N‐Hydroxysuccinimide (NHS)‐ester was then performed as previously described in the whole‐ExM protocol (Sim et al., 2021) with several modifications. Specimens were imaged on a Nikon Eclipse Ti2‐E microscope, with a spinning disk confocal microscope (Dragonfly 200; Andor, Oxford Instruments) equipped with a Zyla 4.2 sCMOS camera (Andor, Oxford Instruments). For all expansion microscopy experiments in this study, the expansion factor determined by landmarks was approximately 4.2‐fold, coincident with the expansion factor determined by gels.

### 4.11 Western blot assay

RIPA buffer (R0278, Sigma‐Aldrich) was used to extract proteins for Western blot. Phosphatase inhibitor cocktail (78420, ThermoFisher) and protease inhibitor cocktail (87786, Thermo Fisher) were added to prevent extra proteolytic and phospholytic activation during cell lysis. The concentration of proteins was adjusted to 10–20 μg measuring in BSA standard curve. All products used in Western blot were purchased from Bio‐Rad. For SDS‐PAGE, concentration of running gel was used in 10% and stacking gel was used in 5%. Nitrocellulose membrane was used to transfer the loaded proteins from running gel and blocked with 5% BSA and 5% skim milk solution in 1% Tween‐20/Tris‐buffered saline (1× TBST) solution for 1 h at RT. Primary antibodies were treated overnight at 4°C, and HRP‐conjugated secondary antibodies were treated at least 2 h at RT. The membranes were washed with TBST solution 3 times for each 10 min between all steps. After all antibody‐conjugated procedures, the membranes were reacted in chemiluminescence by combined a stable peroxide and luminol solution (WestGlow DURA, BioMax), and then, images of detected proteins were captured by X‐ray film imaging by CCD camera in the chemiluminescence image analyzer (ImageQuant LAS 4000 mini, GE Healthcare). Antibodies used in this research were listed in Table S3.

### 4.12 Polymerase chain reaction (PCR)

PCR was initiated by RNA extraction following the Trizol method. Concentration of extracted RNA was measured by nanodrop spectrophotometer (DS‐11, DeNovix). The ratio of absorbance at 260 and 280 nm in all samples was controlled between 1.6 and 2.0. The amount of RNA was adjusted to 300 ng per one sample. Then, cDNA synthesis kit (PB30.31‐10, PCR Biosystems) was used for reverse transcription PCR (RT‐PCR), and sybr green mix (PB20.15‐05, PCR Biosystems) was used in quantitative real‐time PCR (qPCR). RT‐PCR and qPCR were all performed in CFX96 Real‐Time PCR detection system (Bio‐Rad). The sequences of primers used in this research were listed in Table S4.

### 4.13 Analysis of bulk RNA‐Seq data

As total RNA was isolated using Trizol method (detailed in PCR method part), QC validation and total RNA sequencing were all performed by ebiogen (Seoul, Korea). RNA quality was assessed by Agilent 2100 bioanalyzer using RNA 6000 Nano Chip (Agilent Technologies), and RNA quantification was performed using ND‐2000 Spectrophotometer (Thermo). High‐throughput sequencing was performed as single‐end 75 sequencing using NextSeq 500 (Illumina). QuantSeq 3′ mRNA‐Seq reads were aligned to the human reference genome sequence (GRCh38, GENCODE v. 39) by using STAR version 2.7.10a program. The alignment process by STAR was performed with the options of “‐‐runMode alignReads,” “‐‐outSAMtype BAM SortedBy Coordinate,” and “‐‐quantMode TranscriptomeSAM.” After alignments, the gene expression analyses were followed by samtools sort method using BAM‐aligned files sorted by coordinate and differentially expressed gene were determined based on counts from htseq with the options of “‐‐format bam,” “‐‐order pos,” “‐‐mode intersection‐strict,” and “‐‐stranded yes.” To perform between‐sample comparison, we used DEGexp command in DEGseq package by randomly sampled from every possible nucleotide both independently and uniformly (Wang et al., 2009). The output from DEGexp contained log2 fold change and significance of DEG, and were sorted by ECM‐related gene lists which was showed in Table S5. In addition, the other hand, normalization for multi‐sample comparison was performed by edgeR package (Robinson et al., 2010). DGEList objects stored in edgeR were filtered in minimum counts of all samples which we want to compare by log2‐transformation of counts per million (CPM) within calculated scaling factor using trimmed mean of the M‐values (TMM).

### 4.14 Analysis of single‐cell RNA‐Seq data

A published scRNAseq data were downloaded from https://singlecell.broadinstitute.org. scRNAseq data files GSE129788_RAW.tar were used and analyzed with Seurat.

### 4.15 Statistics

Data were presented as the mean with error bar of standard error of the mean (SEM). Levels of significance between samples compared to the standard were determined using Student's *t*‐test. Differences were considered statistically significant with *p*‐value which was auto‐calculated and auto‐displayed in DataGraph for macOS (Visual Data Tools).

## AUTHOR CONTRIBUTIONS

H.M.K., Y.J., I.Y., and P.K. designed research; H.M.K. and S.K. performed the animal experimental research; H.M.K., J.S., and J.‐B.C. performed enhanced confocal imaging for human brain tissue; H.M.K., B.S.M., and T.‐S.K. performed tensile test for collagen vitrified membrane; S.‐H.P. provided human brain tissue; H.M.K. performed all in vitro experiments, data analysis, and statistical tests; H.M.K., S.K., Y.J., and P.K. supported financial funding acquisition; H.M.K. and P.K. wrote the paper with all authors contributing.

## CONFLICT OF INTEREST STATEMENT

The authors declare that they have no conflict of interest.

## Supporting information


Figure S1–S9
Table S1–S5

## Data Availability

The RNA sequencing data are available through NCBI's Gene Expression Omnibus (GEO) under the accession number GSE216298.
