# Supplementary material for: Glycation‐mediated tissue‐level remodeling of brain meningeal membrane by aging
Source: Aging Cell. 2023 Feb 28;22(5):e13805. doi: 10.1111/acel.13805 (PMC10186607; doi:10.1111/acel.13805)
Supplement: Supplementary file 1 — Figure S1–S9 Table S1–S5 [file ACEL-22-e13805-s001.docx]

**Supplementary Figures**


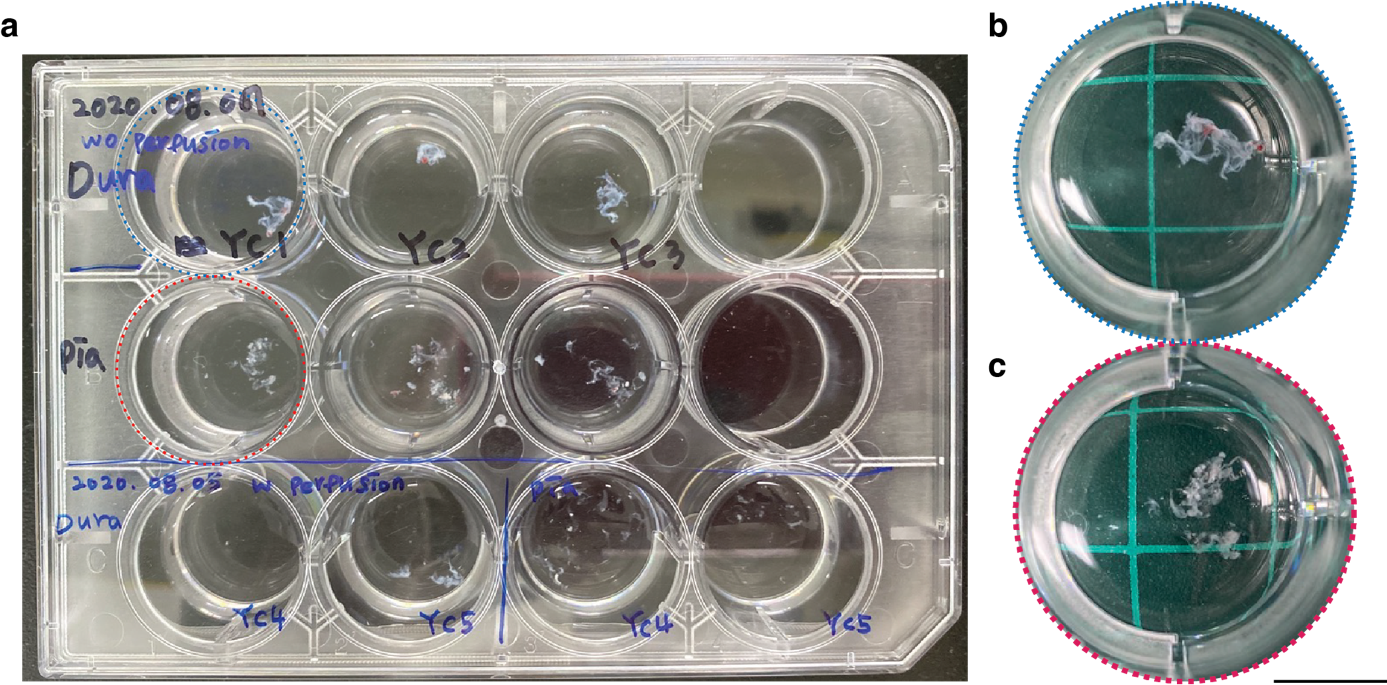


**Figure S1.** Leptomeningeal membranes are separated from dura mater and collected for western blot assay. a) Example photo of separated leptomeningeal membrane from dura mater membrane is showed in young mice brain. ‘Dura’ in photo denotes dura mater membrane, ‘Pia’ in photo denotes leptomeningeal membrane (pia + arachnoid), and ‘YC’ in photo denotes Young Control with the number of mice. b) Magnified image of dura mater membrane from YC1 is represented, and c) magnified image of leptomeningeal membrane from same mice is represented (scale bar, 1 cm).


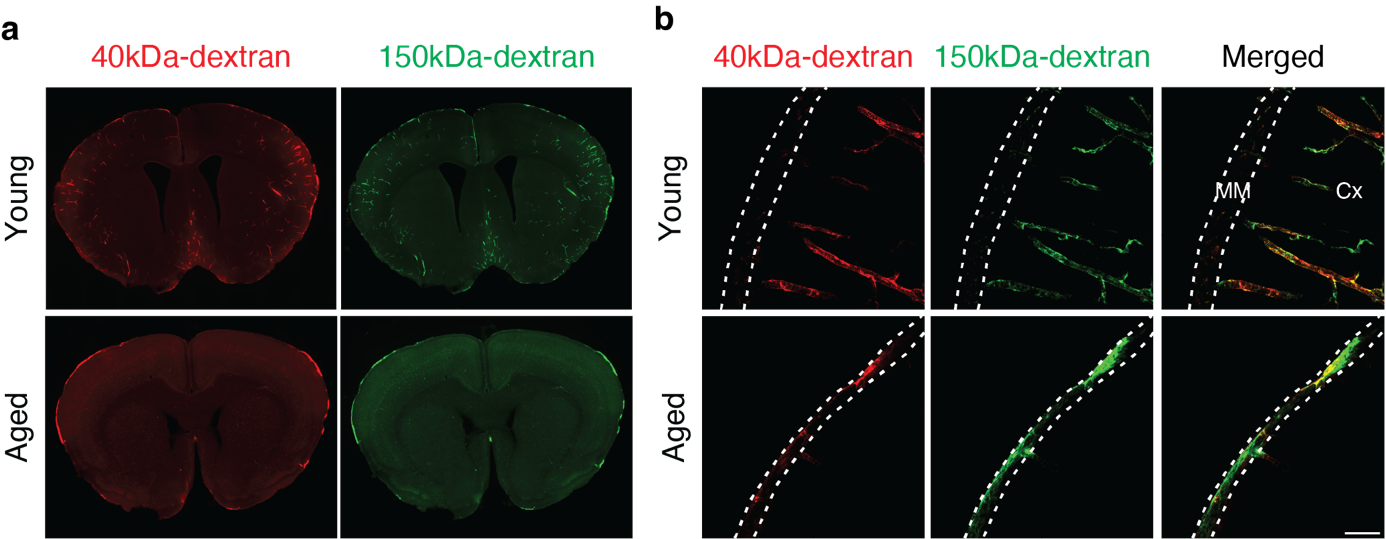


**Figure S2.** CSF-injected fluorescence tracers were blocked to penetrate into the paravascular space in the aged mice brain. a) As we injected fluorescence tracers into CSF, we observed the reduction of molecular penetration through paravascular space in both 40kDa and 150kDa sized macromolecules in the aged mice brain as well as increased molecular adsorption on the meningeal membrane. b) In the magnified images, the blockage of molecular penetration in the aged mice brain is well displayed (scale bar, 100 μm). MM, meningeal membrane. Cx, cortex.


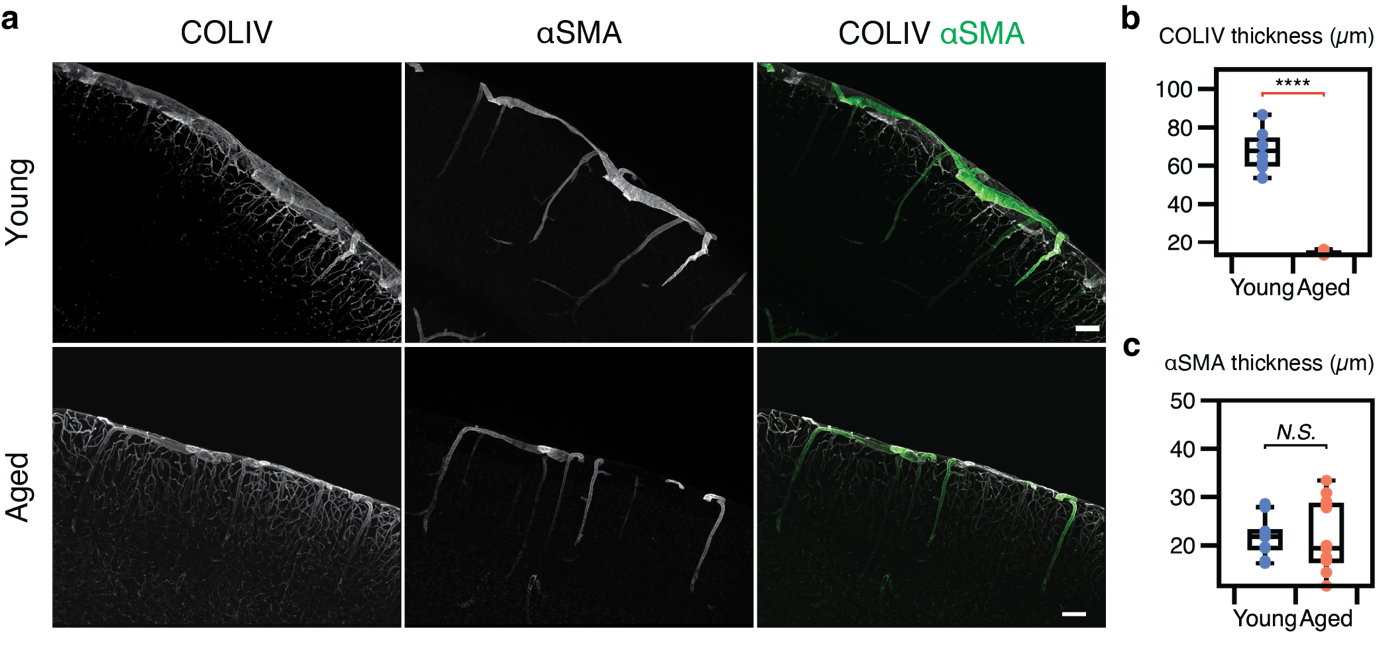


**Figure S3.** Comparing vascular structure in the young and aged meningeal membrane. a) Vascular structures along the meningeal membrane are shown by protein expression of COL4 and αSMA (scale bar, 100 μm). b) The thickness of COL4 protein expression is decreased in aged mice brain, and c) the thickness of αSMA protein expression shows no significant change by aging.


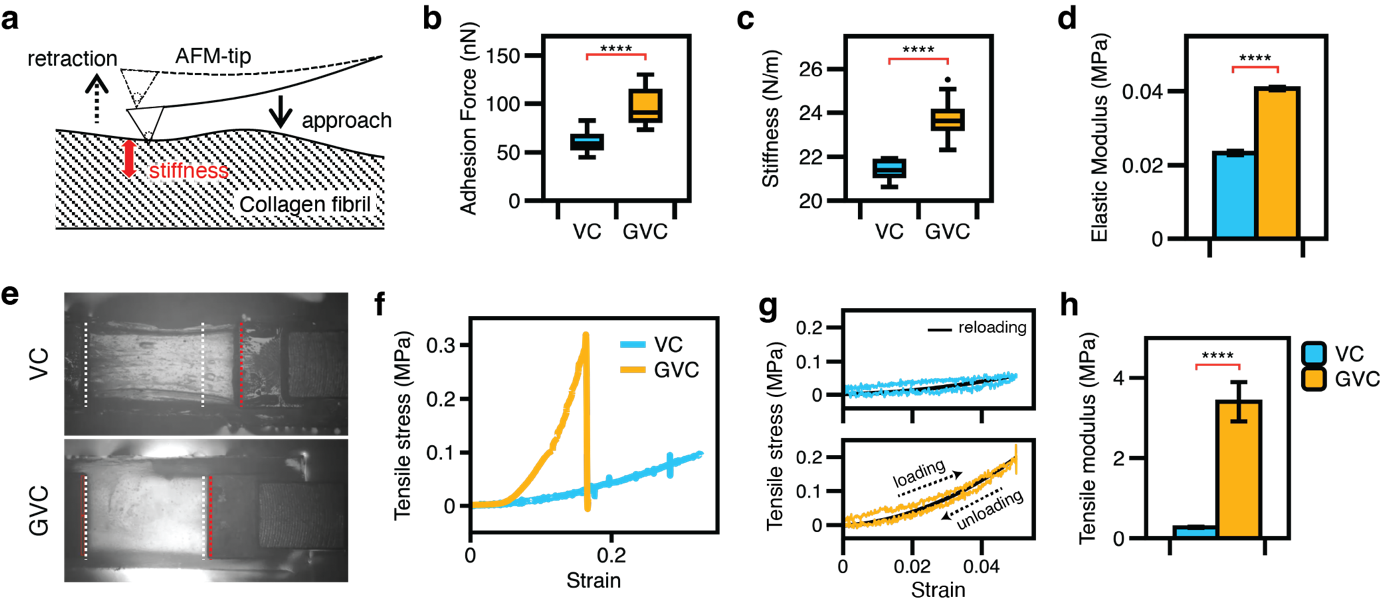


**Figure S4.** Comparing mechanical characterization of collagen membranes by glycation. a) Schematic diagram of fibrillar detection by AFM. b) AFM-detected adhesion force is higher in GVC than VC. c) AFM-detected fibrillar stiffness is also increased in GVC. d) Given shear stress on the bulk membrane, the elastic modulus is also increased in GVC. e) Tensile test on membrane was performed. White dotted lines indicate the initial point for tensile test and red dotted lines indicate a certain point when same tensile stress (0.18 MPa) was given. VC shows more deformation compared to GVC given the same tensile stress. f) Strain-stress curve for tensile test shows GVC gets higher stress than VC under same strain rate. g) From the stress-strain curve in load and unload tensile test, both VC and GVC return to zero load so that we demonstrated that both VC and GVC have elastic properties in the bulk membrane scale. h) Calculating tensile modulus by slope in strain-stress curve, the tensile modulus of GVC is about 12 times higher than that of VC. All data are presented as the mean ± SEM. *p < 0.05, **p < 0.01, ***p < 0.001, ****p < 0.0001.


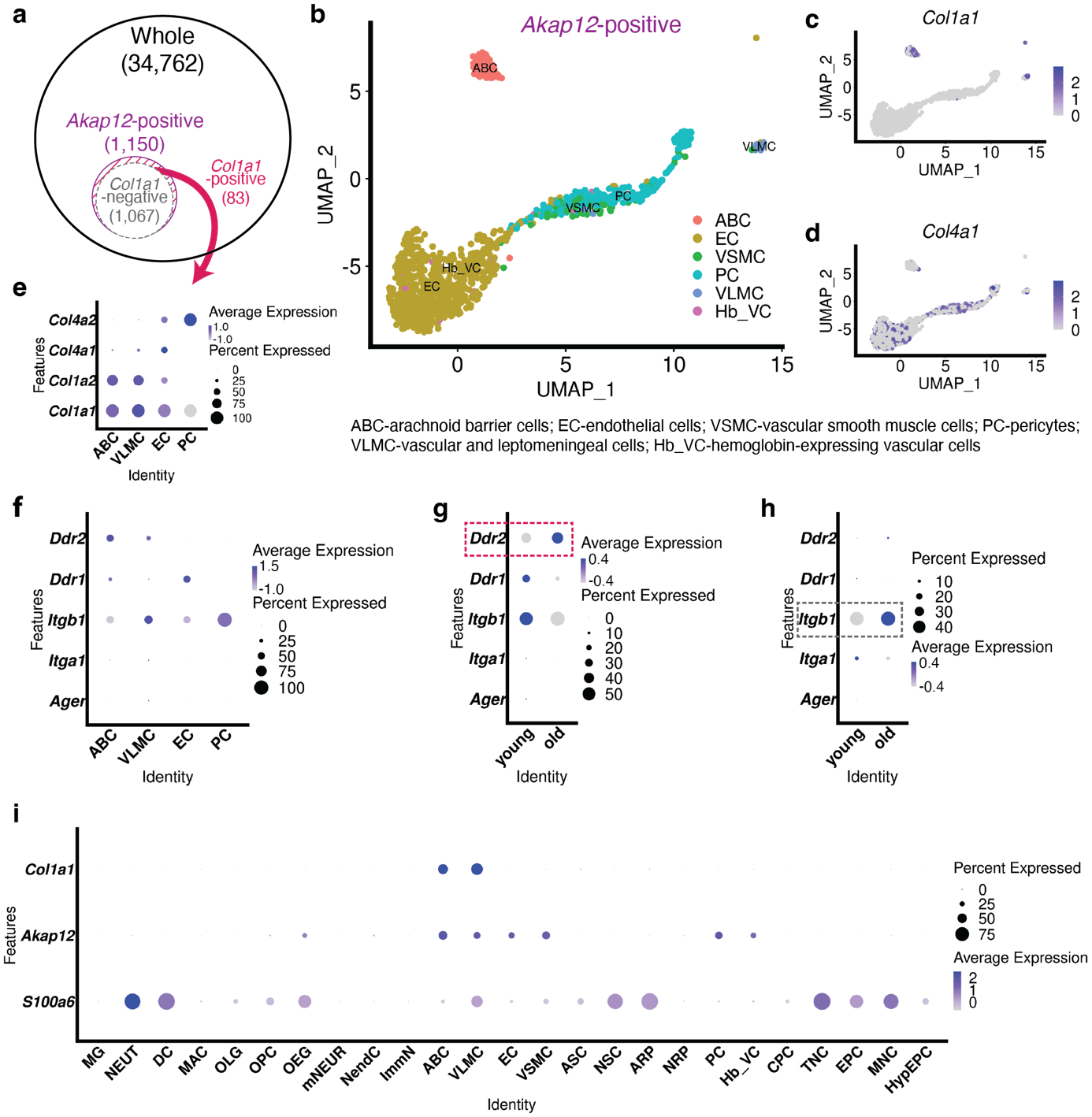


**Figure S5.** Akap12-positive meningeal fibroblast cells have Ddr2-mediated matrix binding instead of Itgb1 by aging. a) There are 1,150 Akap12-positive (Akap12^+^) cells among the whole 34,762 brain cells. Only 83 cells have positive expression of Col1a1 among Akap12^+^ cells. b) Akap12^+^ cells are composed of arachnoid barrier cells (ABC), endothelial cells (EC), vascular smooth muscle cells (VSMC), pericytes (PC), hemoglobin-expressing vascular cells (Hb_VC), and vascular and leptomeningeal cells (VLMC) which are sorted in ^[21]^. c) Particularly, most of ABC and VLMC are Col1a1-positive (Col1a1^+^) cells and d) some of remnants are Col4a1-positive (Col4a1^+^) cells. (e-g) Among both Akap12 and Col1a1 positive cells (Akap12^+^/Col1a1^+^), (e) ABC and VLMC show Col1a1 and Col1a2 expression, not Col4a1 and Col4a2 expression. (f) In the cell-matrix interactive proteins, Akap12^+^/Col1a1^+^ cells mainly bind on the matrix with Itgb1 and Ddr2. They also show Ager (gene encoding RAGE)-negative expression, despite of AGE accumulation along the meningeal membrane. (g) Especially, Akap12^+^/Col1a1^+^ cells show increased Ddr2 expression by aging. (h) Akap12-positive, but Col1a1-negative cells (Akap12^+^/Col1a1^−^) show reversely increased Itgb1 expression by aging. (i) S100a6, one of well-known markers for meningeal fibroblast cells ***^[34]^***, is expressed on the overall brain cells. This brings Akap12 as a representative marker for meningeal fibroblast cells following Col1a1 expression.


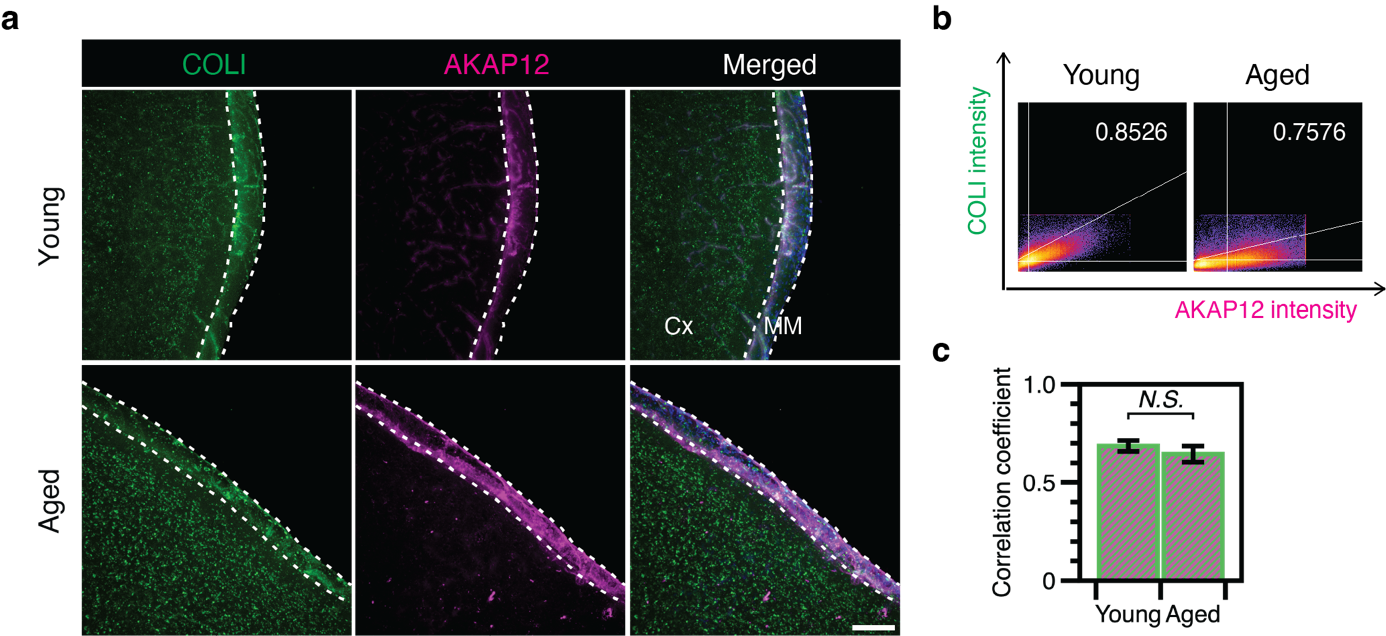


**Figure S6.** COL1-expressing meningeal fibroblast cells have positive correlation with AKAP12 expression. a) We verified Akap12^+^/Col1a1^+^ cells along the meningeal membrane in both young and aged mice brain (scale bar, 100 μm). (b, c) The correlation rate in intensity of COL1 and AKAP12 expression on the meningeal membrane is measured by Fiji. Spearman’s rank correlation value (b) and Pearson’s R value (c) were quantified and they show similar correlation of fluorescence intensity of COL1 and AKAP12 in both young and aged mice brain. MM, meningeal membrane. Cx, cortex.


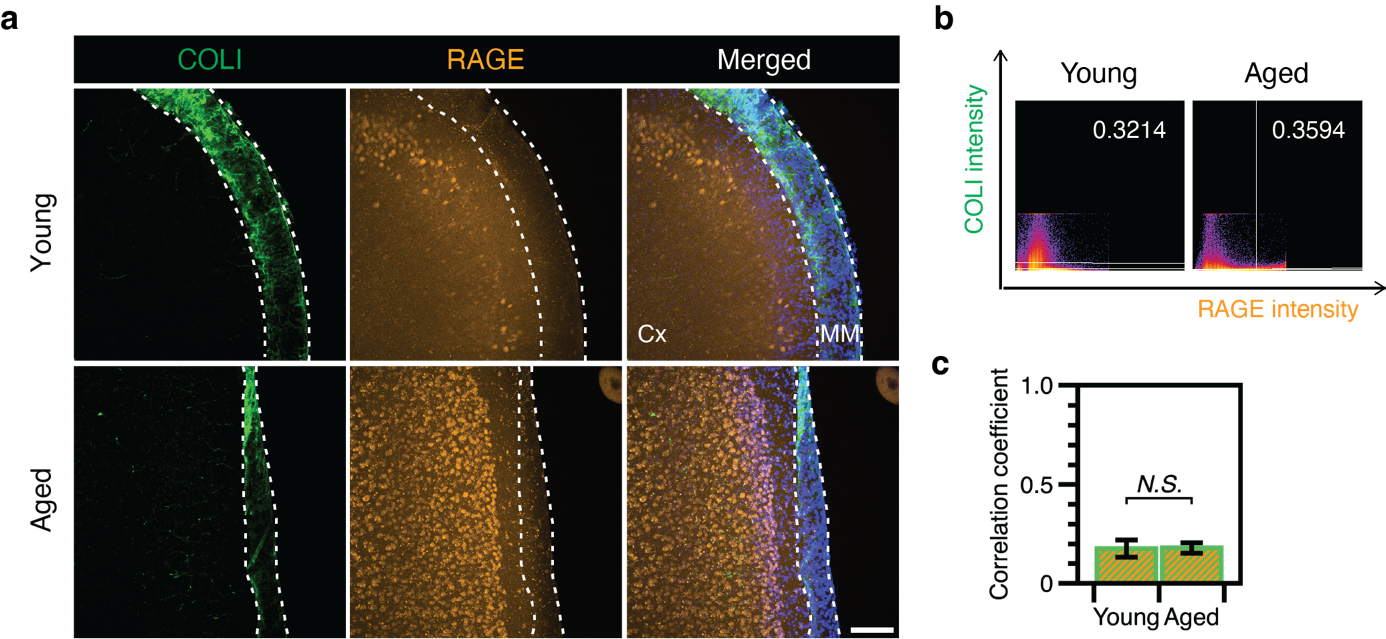


**Figure S7.** COL1-expressing meningeal fibroblast cells have non-positive correlation with RAGE even in aged mice brain. a) We also validated there is less expression of RAGE along the COL1-expressing meningeal membrane in the mice brain (scale bar, 100 μm). (b, c) In both young and aged mice, they have lack of correlation between COL1 and RAGE expression on the meningeal membrane. Spearman’s rank correlation value (b) and Pearson’s R value (c) were quantified by Fiji and they show similar correlation of fluorescence intensity of COL1 and RAGE in both young and aged mice brain. MM, meningeal membrane. Cx, cortex.


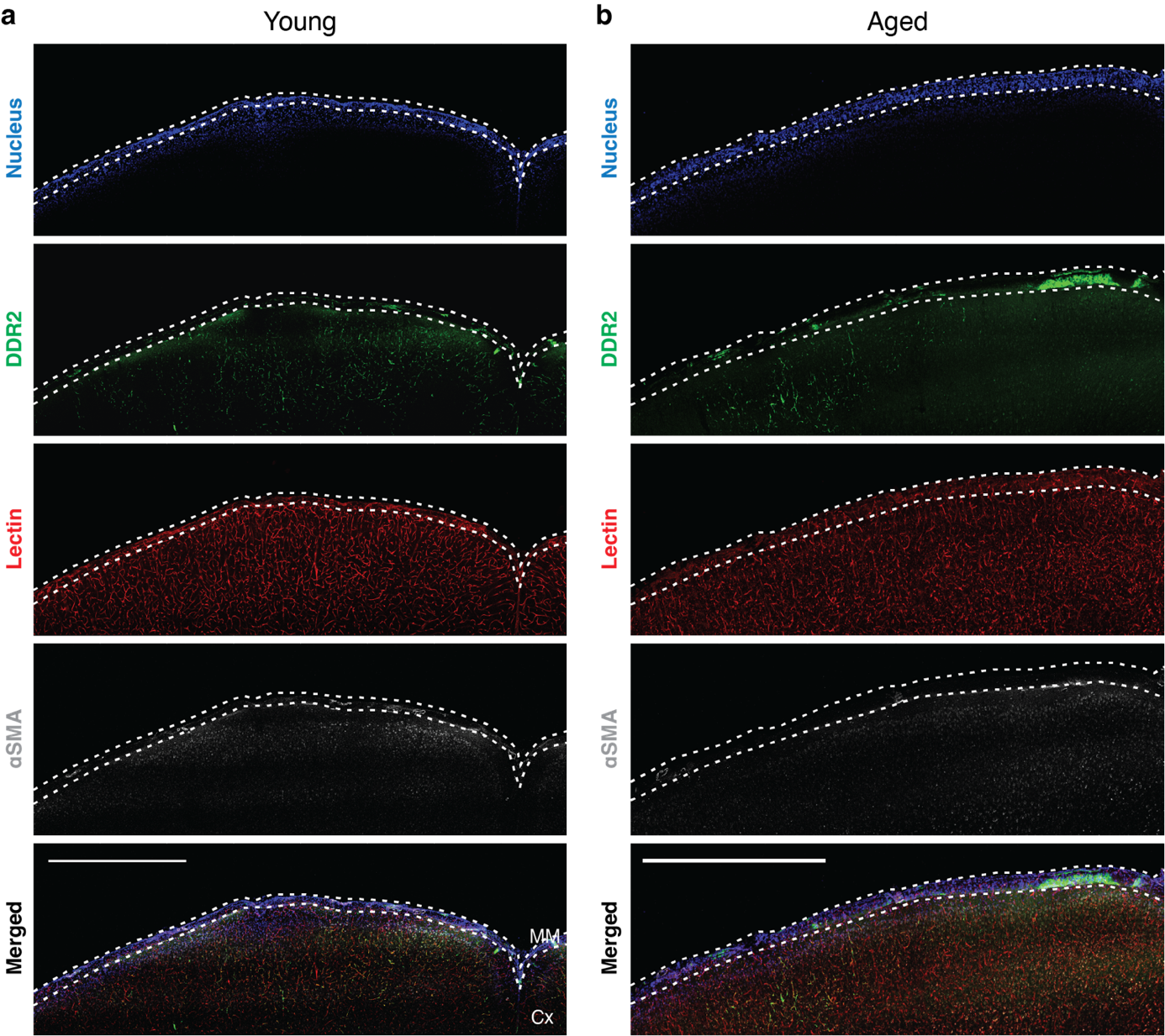


**Fig. S8. Aged mice brain shows widely high expression of DDR2 along the meningeal membrane.** (**A**) In young mice brain, there is less DDR2-positive fluorescence intensity along the meningeal membrane. Compared to histological labeling of blood vessel (lectin, αSMA (alpha-smooth muscle actin)) shows the indistinguishable expression between young and aged mice, remarkably lower expression of DDR2 is shown in young mice brain. (B) In the aged mice brain, it shows high intensity of DDR2-positive fluorescence signals on the meningeal membrane. DDR2-expressed area is also shown along the lectin-labeled blood vessel structure inside the cortex in the aged mice brain (scale bar, 100 μm). MM, meningeal membrane. Cx, cortex.


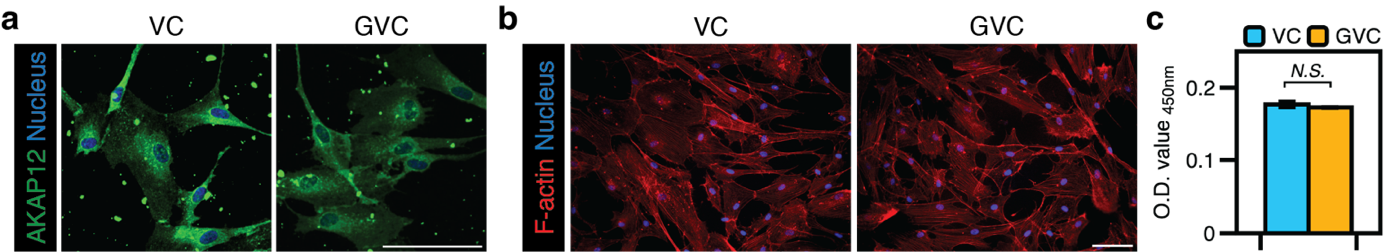


**Figure S9.** Human meningeal fibroblast cells on VC and GVC shows no significant change in morphology and viability. a) Human meningeal fibroblast cells are adapted on both collagen membranes and showed meningeal fibroblast cell marker. There are no significant changes in morphology of meningeal fibroblast cells (b) and viability on the VC and GVC (c). Scale bars are 100 μm. All data are presented as the mean ± SEM.

**Supplementary Tables**

**Table S1:** Patient information for human brain sample

| **No** | **Age** | **Sex** | **Cause of Death** |
| --- | --- | --- | --- |
| #10 | 91 | M | Cerebral infarction |
| #11 | 77 | F | Pneumonia |
| #12 | 81 | M | Pneumonia |
| #18 | 45 | M | Lung Cancer |
| #19 | 72 | M | Septic shock, Hepatitis |

**Table S2:** Antibody lists for immunofluorescence imaging

| **Primary Antibodes** | | **Secondary Antibodes** | |
| --- | --- | --- | --- |
| **Protein** | **Catalog number (dilution)** | **Protein** | **Catalog number (dilution)** |
| DAPI | D9542, Sigma (0.1 ug/ml) | Anti-Mouse FITC | F0257, Sigma (1:500) |
| Phalloidin | P1951, Sigma (2 ug/ml) | Anti-Mouse TRITC | T5393, Sigma (1:500) |
| COLI | ab6308, Abcam (1:500) | Anti-Mouse Alexa Fluor 647 | A21235, Thermo Fisher (1:500) |
|  | ab34710, Abcam (1:500) | Anti-Rabbit FITC | F0382, Sigma (1:500) |
| COL1A1 | sc-293182, Santa Cruz (1:500) | Anti-Rabbit TRITC | T6778, Sigma (1:500) |
| COLIV | ab6586, Abcam (1:400) | Anti-Rabbit Alexa Fluor 647 | A21244, Thermo Fisher (1:500) |
| AGE | abx131709, Abbexa (1:500) |  |  |
| RAGE | sc-365154, Santa Cruz (1:500) |  |  |
|  | ab37647, Abcam (1:500) |  |  |
| ITGB1 | sc-374429, Santa Cruz (1:500) |  |  |
| ITGA2 | ab181548, Abcam (1:500) |  |  |
| VIM | ab137321, Abcam (1:500) |  |  |
| AKAP12 | ab49849, Abcam (1:500) |  |  |
| DDR2 | ab63337, Abcam (1:500) |  |  |
| αSMA | ab5694, Abcam (1:500) |  |  |
| GFAP | ab68428, Abcam (1:500) |  |  |

**Table S3:** Antibody lists for western blot

| **Primary Antibodies** | | |
| --- | --- | --- |
| **Protein** | **Size (kDa)** | **Catalog number (dilution)** |
| GAPDH | 37 | 5174s, Cell Signaling (1:1000) |
| COL1 | 130 | ab34710, abcam (1:1000) |
| COL1A1 | 181 | sc-293182, Santa Cruz (1:1000) |
| ITGB1 | 88 (138) | ab24693, abcam / sc374429, Santa Cruz (1:1000) |
| ACTB | 42 | ab8226, abcam (1:1000) |
| AGE | 68 | ab23722, abcam (1:1000) |
| DDR2 | 97 | ab63337, abcam (1:1000) |
| p-DDR2 | 120-130 | MAB25382, R&D Systems (1:5000) |
| RAGE | 46 | sc-365154, Santa Cruz (1:200) |
| MMP14 | 66 | ab51074, abcam (1:2000) |
| TIMP1 | 23 | sc21734, Santa Cruz (1:200) |
| **Secondary Antibodies** | | |
| **Protein** | | **Catalog number (dilution)** |
| Anti-Mouse IgG (whole molecule)–Peroxidase | | A9044, Sigma (1:2500) |
| Anti-Rabbit IgG (whole molecule)–Peroxidase | | A0545, Sigma (1:2500) |

**Table S4:** Primer lists for PCR

| **Gene** | **Forward sequence (5’ 🡪 3’)** | **Reverse sequence (3’ 🡪 5’)** |
| --- | --- | --- |
| *human GAPDH* | CGGAGTCAACGGATTTGGTCGTAT | AGCCTTCTCCATGGTGGTGAAGAC |
| *human ACTB* | CACCTTCTACAATGAGCTGC | AGGCAGCTCGTAGCTCTTCT |
| *human ITGA2* | GGAACGGGACTTTCGCAT | GGTACTTCGGCTTTCTCATCA |
| *human ITGB1* | CCGCGCGGAAAAGATGAAT | CCACAATTTGGCCCTGCTTG |
| *human VIM* | GCAAAGATTCCACTTTGCGT | GAAATTGCAGGAGGAGATGC |
| *human DDR1* | TGGTGGGCCTGGATGATTTC | GTGGGGAAGCTCTGATTGCT |
| *human DDR2* | AGCGAGTCCAGCATGTTCAA | GCGAAGGGGGAAGATTCGAT |
| *human COL1A1* | TACAGCGTCACTGTCGATGGC | TCAATCACTGTCTTGCCCCAG |
| *human COL1A2* | CACCCAGAGTGGAGCAGTGG | TTCTTGGCTGGGATGTTTTCA |
| *human COL4A1* | CTACGTGCAAGGCAATGAACG | GCAGAACAGGAAGGGCATTGT |
| *human MMP2* | GCGACAAGAAGTATGGCTTC | TGCCAAGGTCAATGTCAGGA |
| *human MMP14* | CAACACTGCCTACGAGAGGA | GTTCTACCTTCAGCTTCTGG |
| *human TIMP2* | GGCGTTTTGCAATGCAGATGTAG | CACAGGAGCCGTCACTTCTCTTG |
| *human RAGE* | AGCGGCTGGAATGGAAACTGAACA | GAAGGGGCAAGGGCACACCATC |

**Table S5:** ECM-related gene lists for RNA-seq analysis

| **Approved symbol** | **Approved name** | **NCBI Gene ID** | **HGNC ID** |
| --- | --- | --- | --- |
| A2M | alpha-2-macroglobulin | 2 | HGNC:7 |
| ACTN1 | actinin alpha 1 | 87 | HGNC:163 |
| ADAM8 | ADAM metallopeptidase domain 8 | 101 | HGNC:215 |
| ADAM10 | ADAM metallopeptidase domain 10 | 102 | HGNC:188 |
| ADAM12 | ADAM metallopeptidase domain 12 | 8038 | HGNC:190 |
| ADAM15 | ADAM metallopeptidase domain 15 | 8751 | HGNC:193 |
| ADAM19 | ADAM metallopeptidase domain 19 | 8728 | HGNC:197 |
| ADAMTS3 | ADAM metallopeptidase with thrombospondin type 1 motif 3 | 9508 | HGNC:219 |
| ADAMTS4 | ADAM metallopeptidase with thrombospondin type 1 motif 4 | 9507 | HGNC:220 |
| ADAMTS5 | ADAM metallopeptidase with thrombospondin type 1 motif 5 | 11096 | HGNC:221 |
| AGRN | agrin | 375790 | HGNC:329 |
| AGT | angiotensinogen | 183 | HGNC:333 |
| AK6 | adenylate kinase 6 | 102157402 | HGNC:49151 |
| APP | amyloid beta precursor protein | 351 | HGNC:620 |
| APPL2 | adaptor protein, phosphotyrosine interacting with PH domain and leucine zipper 2 | 55198 | HGNC:18242 |
| AQP1 | aquaporin 1 (Colton blood group) | 358 | HGNC:633 |
| ATP7A | ATPase copper transporting alpha | 538 | HGNC:869 |
| B4GALT7 | beta-1,4-galactosyltransferase 7 | 11285 | HGNC:930 |
| BABAM1 | BRISC and BRCA1 A complex member 1 | 29086 | HGNC:25008 |
| BARD1 | BRCA1 associated RING domain 1 | 580 | HGNC:952 |
| BCAN | brevican | 63827 | HGNC:23059 |
| BGN | biglycan | 633 | HGNC:1044 |
| BMI1 | BMI1 proto-oncogene, polycomb ring finger | 648 | HGNC:1066 |
| BMP1 | bone morphogenetic protein 1 | 649 | HGNC:1067 |
| BRCA1 | BRCA1 DNA repair associated | 672 | HGNC:1100 |
| BRCC3 | BRCA1/BRCA2-containing complex subunit 3 | 79184 | HGNC:24185 |
| BSG | basigin (Ok blood group) | 682 | HGNC:1116 |
| CAPG | capping actin protein, gelsolin like | 822 | HGNC:1474 |
| CAPN1 | calpain 1 | 823 | HGNC:1476 |
| CAPN2 | calpain 2 | 824 | HGNC:1479 |
| CAPNS1 | calpain small subunit 1 | 826 | HGNC:1481 |
| CAPNS2 | calpain small subunit 2 | 84290 | HGNC:16371 |
| CASK | calcium/calmodulin dependent serine protein kinase | 8573 | HGNC:1497 |
| CCNL1 | cyclin L1 | 57018 | HGNC:20569 |
| CD44 | CD44 molecule (Indian blood group) | 960 | HGNC:1681 |
| CD47 | CD47 molecule | 961 | HGNC:1682 |
| CD74 | CD74 molecule | 972 | HGNC:1697 |
| CD300A | CD300a molecule | 11314 | HGNC:19319 |
| CDC73 | cell division cycle 73 | 79577 | HGNC:16783 |
| CDH1 | cadherin 1 | 999 | HGNC:1748 |
| CDK4 | cyclin dependent kinase 4 | 1019 | HGNC:1773 |
| CDK6 | cyclin dependent kinase 6 | 1021 | HGNC:1777 |
| CDKN1A | cyclin dependent kinase inhibitor 1A | 1026 | HGNC:1784 |
| CENPC | centromere protein C | 1060 | HGNC:1854 |
| CHADL | chondroadherin like | 150356 | HGNC:25165 |
| CLASP1 | cytoplasmic linker associated protein 1 | 23332 | HGNC:17088 |
| CLASP2 | cytoplasmic linker associated protein 2 | 23122 | HGNC:17078 |
| CMA1 | chymase 1 | 1215 | HGNC:2097 |
| COL1A1 | collagen type I alpha 1 chain | 1277 | HGNC:2197 |
| COL1A2 | collagen type I alpha 2 chain | 1278 | HGNC:2198 |
| COL2A1 | collagen type II alpha 1 chain | 1280 | HGNC:2200 |
| COL3A1 | collagen type III alpha 1 chain | 1281 | HGNC:2201 |
| COL4A1 | collagen type IV alpha 1 chain | 1282 | HGNC:2202 |
| COL4A2 | collagen type IV alpha 2 chain | 1284 | HGNC:2203 |
| COL4A3 | collagen type IV alpha 3 chain | 1285 | HGNC:2204 |
| COL4A4 | collagen type IV alpha 4 chain | 1286 | HGNC:2206 |
| COL4A5 | collagen type IV alpha 5 chain | 1287 | HGNC:2207 |
| COL4A6 | collagen type IV alpha 6 chain | 1288 | HGNC:2208 |
| COL5A1 | collagen type V alpha 1 chain | 1289 | HGNC:2209 |
| COL5A2 | collagen type V alpha 2 chain | 1290 | HGNC:2210 |
| COL5A3 | collagen type V alpha 3 chain | 50509 | HGNC:14864 |
| COL6A1 | collagen type VI alpha 1 chain | 1291 | HGNC:2211 |
| COL6A2 | collagen type VI alpha 2 chain | 1292 | HGNC:2212 |
| COL6A3 | collagen type VI alpha 3 chain | 1293 | HGNC:2213 |
| COL7A1 | collagen type VII alpha 1 chain | 1294 | HGNC:2214 |
| COL8A1 | collagen type VIII alpha 1 chain | 1295 | HGNC:2215 |
| COL8A2 | collagen type VIII alpha 2 chain | 1296 | HGNC:2216 |
| COL9A1 | collagen type IX alpha 1 chain | 1297 | HGNC:2217 |
| COL9A2 | collagen type IX alpha 2 chain | 1298 | HGNC:2218 |
| COL9A3 | collagen type IX alpha 3 chain | 1299 | HGNC:2219 |
| COL10A1 | collagen type X alpha 1 chain | 1300 | HGNC:2185 |
| COL11A1 | collagen type XI alpha 1 chain | 1301 | HGNC:2186 |
| COL11A2 | collagen type XI alpha 2 chain | 1302 | HGNC:2187 |
| COL12A1 | collagen type XII alpha 1 chain | 1303 | HGNC:2188 |
| COL13A1 | collagen type XIII alpha 1 chain | 1305 | HGNC:2190 |
| COL14A1 | collagen type XIV alpha 1 chain | 7373 | HGNC:2191 |
| COL16A1 | collagen type XVI alpha 1 chain | 1307 | HGNC:2193 |
| COL18A1 | collagen type XVIII alpha 1 chain | 80781 | HGNC:2195 |
| COL19A1 | collagen type XIX alpha 1 chain | 1310 | HGNC:2196 |
| COMP | cartilage oligomeric matrix protein | 1311 | HGNC:2227 |
| CREB3L1 | cAMP responsive element binding protein 3 like 1 | 90993 | HGNC:18856 |
| CSGALNACT1 | chondroitin sulfate N-acetylgalactosaminyltransferase 1 | 55790 | HGNC:24290 |
| CST3 | cystatin C | 1471 | HGNC:2475 |
| CTNNB1 | catenin beta 1 | 1499 | HGNC:2514 |
| CTRB1 | chymotrypsinogen B1 | 1504 | HGNC:2521 |
| CTRB2 | chymotrypsinogen B2 | 440387 | HGNC:2522 |
| CTSG | cathepsin G | 1511 | HGNC:2532 |
| CTSK | cathepsin K | 1513 | HGNC:2536 |
| CTSL | cathepsin L | 1514 | HGNC:2537 |
| CTSS | cathepsin S | 1520 | HGNC:2545 |
| CTSV | cathepsin V | 1515 | HGNC:2538 |
| CYP1B1 | cytochrome P450 family 1 subfamily B member 1 | 1545 | HGNC:2597 |
| DAB2IP | DAB2 interacting protein | 153090 | HGNC:17294 |
| DAG1 | dystroglycan 1 | 1605 | HGNC:2666 |
| DCN | decorin | 1634 | HGNC:2705 |
| DDR1 | discoidin domain receptor tyrosine kinase 1 | 780 | HGNC:2730 |
| DDR2 | discoidin domain receptor tyrosine kinase 2 | 4921 | HGNC:2731 |
| DDRGK1 | DDRGK domain containing 1 | 65992 | HGNC:16110 |
| DMD | dystrophin | 1756 | HGNC:2928 |
| DMP1 | dentin matrix acidic phosphoprotein 1 | 1758 | HGNC:2932 |
| DPP4 | dipeptidyl peptidase 4 | 1803 | HGNC:3009 |
| DPT | dermatopontin | 1805 | HGNC:3011 |
| DSPP | dentin sialophosphoprotein | 1834 | HGNC:3054 |
| E2F1 | E2F transcription factor 1 | 1869 | HGNC:3113 |
| EIF3A | eukaryotic translation initiation factor 3 subunit A | 8661 | HGNC:3271 |
| ELANE | elastase, neutrophil expressed | 1991 | HGNC:3309 |
| ELN | elastin | 2006 | HGNC:3327 |
| EMD | emerin | 2010 | HGNC:3331 |
| EP300 | E1A binding protein p300 | 2033 | HGNC:3373 |
| EREG | epiregulin | 2069 | HGNC:3443 |
| EXOC8 | exocyst complex component 8 | 149371 | HGNC:24659 |
| F11R | F11 receptor | 50848 | HGNC:14685 |
| FAP | fibroblast activation protein alpha | 2191 | HGNC:3590 |
| FBLN5 | fibulin 5 | 10516 | HGNC:3602 |
| FBN1 | fibrillin 1 | 2200 | HGNC:3603 |
| FBN2 | fibrillin 2 | 2201 | HGNC:3604 |
| FGA | fibrinogen alpha chain | 2243 | HGNC:3661 |
| FGB | fibrinogen beta chain | 2244 | HGNC:3662 |
| FGF2 | fibroblast growth factor 2 | 2247 | HGNC:3676 |
| FGFR4 | fibroblast growth factor receptor 4 | 2264 | HGNC:3691 |
| FGG | fibrinogen gamma chain | 2266 | HGNC:3694 |
| FLOT1 | flotillin 1 | 10211 | HGNC:3757 |
| FLRT2 | fibronectin leucine rich transmembrane protein 2 | 23768 | HGNC:3761 |
| FN1 | fibronectin 1 | 2335 | HGNC:3778 |
| FSCN1 | fascin actin-bundling protein 1 | 6624 | HGNC:11148 |
| FTH1 | ferritin heavy chain 1 | 2495 | HGNC:3976 |
| FURIN | furin, paired basic amino acid cleaving enzyme | 5045 | HGNC:8568 |
| GAS6 | growth arrest specific 6 | 2621 | HGNC:4168 |
| GPM6B | glycoprotein M6B | 2824 | HGNC:4461 |
| GREM1 | gremlin 1, DAN family BMP antagonist | 26585 | HGNC:2001 |
| GSN | gelsolin | 2934 | HGNC:4620 |
| GSTP1 | glutathione S-transferase pi 1 | 2950 | HGNC:4638 |
| H2AX | H2A.X variant histone | 3014 | HGNC:4739 |
| HAPLN1 | hyaluronan and proteoglycan link protein 1 | 1404 | HGNC:2380 |
| HAS1 | hyaluronan synthase 1 | 3036 | HGNC:4818 |
| HAS2 | hyaluronan synthase 2 | 3037 | HGNC:4819 |
| HAS3 | hyaluronan synthase 3 | 3038 | HGNC:4820 |
| HPN | hepsin | 3249 | HGNC:5155 |
| HSPG2 | heparan sulfate proteoglycan 2 | 3339 | HGNC:5273 |
| HTRA1 | HtrA serine peptidase 1 | 5654 | HGNC:9476 |
| HTT | huntingtin | 3064 | HGNC:4851 |
| IBSP | integrin binding sialoprotein | 3381 | HGNC:5341 |
| ICAM1 | intercellular adhesion molecule 1 | 3383 | HGNC:5344 |
| ICAM2 | intercellular adhesion molecule 2 | 3384 | HGNC:5345 |
| ICAM3 | intercellular adhesion molecule 3 | 3385 | HGNC:5346 |
| ICAM4 | intercellular adhesion molecule 4 (Landsteiner-Wiener blood group) | 3386 | HGNC:5347 |
| ICAM5 | intercellular adhesion molecule 5 | 7087 | HGNC:5348 |
| IGF1 | insulin like growth factor 1 | 3479 | HGNC:5464 |
| ITGA1 | integrin subunit alpha 1 | 3672 | HGNC:6134 |
| ITGA2 | integrin subunit alpha 2 | 3673 | HGNC:6137 |
| ITGA2B | integrin subunit alpha 2b | 3674 | HGNC:6138 |
| ITGA3 | integrin subunit alpha 3 | 3675 | HGNC:6139 |
| ITGA4 | integrin subunit alpha 4 | 3676 | HGNC:6140 |
| ITGA5 | integrin subunit alpha 5 | 3678 | HGNC:6141 |
| ITGA6 | integrin subunit alpha 6 |  | HGNC:6142 |
| ITGA7 | integrin subunit alpha 7 | 3679 | HGNC:6143 |
| ITGA8 | integrin subunit alpha 8 | 8516 | HGNC:6144 |
| ITGA9 | integrin subunit alpha 9 | 3680 | HGNC:6145 |
| ITGA10 | integrin subunit alpha 10 | 8515 | HGNC:6135 |
| ITGA11 | integrin subunit alpha 11 | 22801 | HGNC:6136 |
| ITGAD | integrin subunit alpha D | 3681 | HGNC:6146 |
| ITGAE | integrin subunit alpha E | 3682 | HGNC:6147 |
| ITGAL | integrin subunit alpha L | 3683 | HGNC:6148 |
| ITGAM | integrin subunit alpha M | 3684 | HGNC:6149 |
| ITGAV | integrin subunit alpha V | 3685 | HGNC:6150 |
| ITGAX | integrin subunit alpha X | 3687 | HGNC:6152 |
| ITGB1 | integrin subunit beta 1 | 3688 | HGNC:6153 |
| ITGB2 | integrin subunit beta 2 | 3689 | HGNC:6155 |
| ITGB3 | integrin subunit beta 3 | 3690 | HGNC:6156 |
| ITGB4 | integrin subunit beta 4 | 3691 | HGNC:6158 |
| ITGB5 | integrin subunit beta 5 | 3693 | HGNC:6160 |
| ITGB6 | integrin subunit beta 6 | 3694 | HGNC:6161 |
| ITGB7 | integrin subunit beta 7 | 3695 | HGNC:6162 |
| ITGB8 | integrin subunit beta 8 | 3696 | HGNC:6163 |
| JAM2 | junctional adhesion molecule 2 | 58494 | HGNC:14686 |
| JAM3 | junctional adhesion molecule 3 | 83700 | HGNC:15532 |
| KAT2B | lysine acetyltransferase 2B | 8850 | HGNC:8638 |
| KDR | kinase insert domain receptor | 3791 | HGNC:6307 |
| KIF9 | kinesin family member 9 | 64147 | HGNC:16666 |
| KLK2 | kallikrein related peptidase 2 | 3817 | HGNC:6363 |
| KLK7 | kallikrein related peptidase 7 | 5650 | HGNC:6368 |
| KLKB1 | kallikrein B1 | 3818 | HGNC:6371 |
| LAMA1 | laminin subunit alpha 1 | 284217 | HGNC:6481 |
| LAMA2 | laminin subunit alpha 2 | 3908 | HGNC:6482 |
| LAMA3 | laminin subunit alpha 3 | 3909 | HGNC:6483 |
| LAMA4 | laminin subunit alpha 4 | 3910 | HGNC:6484 |
| LAMA5 | laminin subunit alpha 5 | 3911 | HGNC:6485 |
| LAMB1 | laminin subunit beta 1 | 3912 | HGNC:6486 |
| LAMB2 | laminin subunit beta 2 | 3913 | HGNC:6487 |
| LAMB3 | laminin subunit beta 3 | 3914 | HGNC:6490 |
| LAMC1 | laminin subunit gamma 1 | 3915 | HGNC:6492 |
| LAMC2 | laminin subunit gamma 2 | 3918 | HGNC:6493 |
| LAMC3 | laminin subunit gamma 3 | 10319 | HGNC:6494 |
| LCP1 | lymphocyte cytosolic protein 1 | 3936 | HGNC:6528 |
| LHFPL4 | LHFPL tetraspan subfamily member 4 | 375323 | HGNC:29568 |
| LIG4 | DNA ligase 4 | 3981 | HGNC:6601 |
| LOX | lysyl oxidase | 4015 | HGNC:6664 |
| LOXL1 | lysyl oxidase like 1 | 4016 | HGNC:6665 |
| LOXL2 | lysyl oxidase like 2 | 4017 | HGNC:6666 |
| LOXL3 | lysyl oxidase like 3 | 84695 | HGNC:13869 |
| LRP1 | LDL receptor related protein 1 | 4035 | HGNC:6692 |
| LUM | lumican | 4060 | HGNC:6724 |
| MADCAM1 | mucosal vascular addressin cell adhesion molecule 1 | 8174 | HGNC:6765 |
| MATN1 | matrilin 1 | 4146 | HGNC:6907 |
| MATN3 | matrilin 3 | 4148 | HGNC:6909 |
| MATN4 | matrilin 4 | 8785 | HGNC:6910 |
| MBP | myelin basic protein | 4155 | HGNC:6925 |
| MED25 | mediator complex subunit 25 | 81857 | HGNC:28845 |
| MFAP2 | microfibril associated protein 2 | 4237 | HGNC:7033 |
| MFAP4 | microfibril associated protein 4 | 4239 | HGNC:7035 |
| MFAP5 | microfibril associated protein 5 | 8076 | HGNC:29673 |
| MIF | macrophage migration inhibitory factor | 4282 | HGNC:7097 |
| MMP1 | matrix metallopeptidase 1 | 4312 | HGNC:7155 |
| MMP2 | matrix metallopeptidase 2 | 4313 | HGNC:7166 |
| MMP3 | matrix metallopeptidase 3 | 4314 | HGNC:7173 |
| MMP7 | matrix metallopeptidase 7 | 4316 | HGNC:7174 |
| MMP8 | matrix metallopeptidase 8 | 4317 | HGNC:7175 |
| MMP9 | matrix metallopeptidase 9 | 4318 | HGNC:7176 |
| MMP10 | matrix metallopeptidase 10 | 4319 | HGNC:7156 |
| MMP11 | matrix metallopeptidase 11 | 4320 | HGNC:7157 |
| MMP12 | matrix metallopeptidase 12 | 4321 | HGNC:7158 |
| MMP13 | matrix metallopeptidase 13 | 4322 | HGNC:7159 |
| MMP14 | matrix metallopeptidase 14 | 4323 | HGNC:7160 |
| MMP15 | matrix metallopeptidase 15 | 4324 | HGNC:7161 |
| MMP16 | matrix metallopeptidase 16 | 4325 | HGNC:7162 |
| MMP17 | matrix metallopeptidase 17 | 4326 | HGNC:7163 |
| MMP19 | matrix metallopeptidase 19 | 4327 | HGNC:7165 |
| MMP21 | matrix metallopeptidase 21 | 118856 | HGNC:14357 |
| MMP24 | matrix metallopeptidase 24 | 10893 | HGNC:7172 |
| MMP25 | matrix metallopeptidase 25 | 64386 | HGNC:14246 |
| MORC3 | MORC family CW-type zinc finger 3 | 23515 | HGNC:23572 |
| MPV17 | mitochondrial inner membrane protein MPV17 | 4358 | HGNC:7224 |
| MYC | MYC proto-oncogene, bHLH transcription factor | 4609 | HGNC:7553 |
| MYH11 | myosin heavy chain 11 | 4629 | HGNC:7569 |
| MYO1E | myosin IE | 4643 | HGNC:7599 |
| MYSM1 | Myb like, SWIRM and MPN domains 1 | 114803 | HGNC:29401 |
| NCAN | neurocan | 1463 | HGNC:2465 |
| NDNF | neuron derived neurotrophic factor | 79625 | HGNC:26256 |
| NDUFS4 | NADH:ubiquinone oxidoreductase subunit S4 | 4724 | HGNC:7711 |
| NET1 | neuroepithelial cell transforming 1 | 10276 | HGNC:14592 |
| NF1 | neurofibromin 1 | 4763 | HGNC:7765 |
| NID1 | nidogen 1 | 4811 | HGNC:7821 |
| NID2 | nidogen 2 | 22795 | HGNC:13389 |
| NLRC3 | NLR family CARD domain containing 3 | 197358 | HGNC:29889 |
| NLRP3 | NLR family pyrin domain containing 3 | 114548 | HGNC:16400 |
| NOTCH1 | notch receptor 1 | 4851 | HGNC:7881 |
| NOXO1 | NADPH oxidase organizer 1 |  | HGNC:19404 |
| NPHS1 | NPHS1 adhesion molecule, nephrin | 4868 | HGNC:7908 |
| NPNT | nephronectin | 255743 | HGNC:27405 |
| NRXN1 | neurexin 1 | 9378 | HGNC:8008 |
| NTN4 | netrin 4 | 59277 | HGNC:13658 |
| OPTC | opticin | 26254 | HGNC:8158 |
| PARP10 | poly(ADP-ribose) polymerase family member 10 | 84875 | HGNC:25895 |
| PDGFA | platelet derived growth factor subunit A | 5154 | HGNC:8799 |
| PDGFB | platelet derived growth factor subunit B | 5155 | HGNC:8800 |
| PDGFC | platelet derived growth factor C | 56034 | HGNC:8801 |
| PDGFRA | platelet derived growth factor receptor alpha | 5156 | HGNC:8803 |
| PDPN | podoplanin | 10630 | HGNC:29602 |
| PEX2 | peroxisomal biogenesis factor 2 | 5828 | HGNC:9717 |
| PHLDB1 | pleckstrin homology like domain family B member 1 | 23187 | HGNC:23697 |
| PHLDB2 | pleckstrin homology like domain family B member 2 | 90102 | HGNC:29573 |
| PICALM | phosphatidylinositol binding clathrin assembly protein | 8301 | HGNC:15514 |
| PLA2G1B | phospholipase A2 group IB | 5319 | HGNC:9030 |
| PLG | plasminogen | 5340 | HGNC:9071 |
| POSTN | periostin | 10631 | HGNC:16953 |
| PRKCA | protein kinase C alpha | 5578 | HGNC:9393 |
| PRSS1 | serine protease 1 | 5644 | HGNC:9475 |
| PRSS2 | serine protease 2 | 5645 | HGNC:9483 |
| PSMD14 | proteasome 26S subunit, non-ATPase 14 | 10213 | HGNC:16889 |
| PXDN | peroxidasin | 7837 | HGNC:14966 |
| RAMP2 | receptor activity modifying protein 2 | 10266 | HGNC:9844 |
| RECK | reversion inducing cysteine rich protein with kazal motifs | 8434 | HGNC:11345 |
| RGCC | regulator of cell cycle | 28984 | HGNC:20369 |
| RPS27A | ribosomal protein S27a | 6233 | HGNC:10417 |
| S100A6 | S100 calcium binding protein A6 | 6277 | HGNC:10496 |
| SCUBE1 | signal peptide, CUB domain and EGF like domain containing 1 | 80274 | HGNC:13441 |
| SCUBE3 | signal peptide, CUB domain and EGF like domain containing 3 | 222663 | HGNC:13655 |
| SCX | scleraxis bHLH transcription factor | 642658 | HGNC:32322 |
| SDC1 | syndecan 1 | 6382 | HGNC:10658 |
| SDC2 | syndecan 2 | 6383 | HGNC:10659 |
| SDC3 | syndecan 3 | 9672 | HGNC:10660 |
| SDC4 | syndecan 4 | 6385 | HGNC:10661 |
| SERPINE1 | serpin family E member 1 | 5054 | HGNC:8583 |
| SERPINF2 | serpin family F member 2 | 5345 | HGNC:9075 |
| SERPINH1 | serpin family H member 1 | 871 | HGNC:1546 |
| SFRP1 | secreted frizzled related protein 1 | 6422 | HGNC:10776 |
| SGK2 | serum/glucocorticoid regulated kinase 2 | 10110 | HGNC:13900 |
| SH3PXD2A | SH3 and PX domains 2A | 9644 | HGNC:23664 |
| SH3PXD2B | SH3 and PX domains 2B | 285590 | HGNC:29242 |
| SKI | SKI proto-oncogene | 6497 | HGNC:10896 |
| SLC4A5 | solute carrier family 4 member 5 | 57835 | HGNC:18168 |
| SMAD3 | SMAD family member 3 | 4088 | HGNC:6769 |
| SORL1 | sortilin related receptor 1 | 6653 | HGNC:11185 |
| SPARC | secreted protein acidic and cysteine rich | 6678 | HGNC:11219 |
| SPHK1 | sphingosine kinase 1 | 8877 | HGNC:11240 |
| SPINK5 | serine peptidase inhibitor Kazal type 5 | 11005 | HGNC:15464 |
| SPOCK2 | SPARC (osteonectin), cwcv and kazal like domains proteoglycan 2 | 9806 | HGNC:13564 |
| SPOCK3 | SPARC (osteonectin), cwcv and kazal like domains proteoglycan 3 | 50859 | HGNC:13565 |
| SPP1 | secreted phosphoprotein 1 | 6696 | HGNC:11255 |
| STAM | signal transducing adaptor molecule | 8027 | HGNC:11357 |
| STAMBP | STAM binding protein | 10617 | HGNC:16950 |
| STAMBPL1 | STAM binding protein like 1 | 57559 | HGNC:24105 |
| STAT3 | signal transducer and activator of transcription 3 | 6774 | HGNC:11364 |
| SULF1 | sulfatase 1 | 23213 | HGNC:20391 |
| SULF2 | sulfatase 2 | 55959 | HGNC:20392 |
| TGFB1 | transforming growth factor beta 1 | 7040 | HGNC:11766 |
| TGFB2 | transforming growth factor beta 2 | 7042 | HGNC:11768 |
| TGFBI | transforming growth factor beta induced | 7045 | HGNC:11771 |
| TGFBR1 | transforming growth factor beta receptor 1 | 7046 | HGNC:11772 |
| THBS1 | thrombospondin 1 | 7057 | HGNC:11785 |
| TIMP1 | TIMP metallopeptidase inhibitor 1 | 7076 | HGNC:11820 |
| TIMP2 | TIMP metallopeptidase inhibitor 2 | 7077 | HGNC:11821 |
| TIMP3 | TIMP metallopeptidase inhibitor 3 | 7078 | HGNC:11822 |
| TLL1 | tolloid like 1 | 7092 | HGNC:11843 |
| TLL2 | tolloid like 2 | 7093 | HGNC:11844 |
| TMPRSS6 | transmembrane serine protease 6 | 164656 | HGNC:16517 |
| TNC | tenascin C | 3371 | HGNC:5318 |
| TNR | tenascin R | 7143 | HGNC:11953 |
| TNXB | tenascin XB | 7148 | HGNC:11976 |
| TP53 | tumor protein p53 | 7157 | HGNC:11998 |
| TPSAB1 | tryptase alpha/beta 1 | 7177 | HGNC:12019 |
| TRAPPC4 | trafficking protein particle complex subunit 4 | 51399 | HGNC:19943 |
| TRIM32 | tripartite motif containing 32 | 22954 | HGNC:16380 |
| TTR | transthyretin | 7276 | HGNC:12405 |
| UBA52 | ubiquitin A-52 residue ribosomal protein fusion product 1 | 7311 | HGNC:12458 |
| UBB | ubiquitin B | 7314 | HGNC:12463 |
| UBC | ubiquitin C | 7316 | HGNC:12468 |
| UIMC1 | ubiquitin interaction motif containing 1 | 51720 | HGNC:30298 |
| VAT1 | vesicle amine transport 1 | 10493 | HGNC:16919 |
| VCAM1 | vascular cell adhesion molecule 1 | 7412 | HGNC:12663 |
| VCAN | versican | 1462 | HGNC:2464 |
| VTN | vitronectin | 7448 | HGNC:12724 |
| VWF | von Willebrand factor | 7450 | HGNC:12726 |
| WNT1 | Wnt family member 1 | 7471 | HGNC:12774 |
| WNT2 | Wnt family member 2 | 7472 | HGNC:12780 |
| WNT5A | Wnt family member 5A | 7474 | HGNC:12784 |
| WT1 | WT1 transcription factor | 7490 | HGNC:12796 |
